# KLF7 promotes colon adenocarcinoma progression through the PDGFB signaling pathway

**DOI:** 10.7150/ijbs.86385

**Published:** 2024-01-01

**Authors:** Zhicheng Zhang, Xiaochen Jiang, Kai Li, Shupei Qiao, Mengmeng Li, Yu Mei, Lixian Ding, Qiang Lv, Yike Ding, Yunhan Zhao, Guixiang Lv, Gang Tan, Huanjie Yang, Guodong Li, Xu Gao, Ming Liu

**Affiliations:** 1 Department of General Surgery, The Fourth Affiliated Hospital of Harbin Medical University, Harbin,150001, China.; 2 Bio-Bank of Department of General Surgery, The Fourth Affiliated Hospital of Harbin Medical University, Harbin, 150001, China.; 3 Department of Biochemistry and Molecular Biology, Harbin Medical University, Harbin, 150001, China.; 4 Editorial Board of Harbin Medical University, Harbin Medical University, Harbin 150001, China.; 5 School of Medicine and Health, Harbin Institute of Technology, Harbin, 150001, China.; 6 Heilongjiang Key Laboratory of Children Development and Genetic Research, Harbin Medical University, Harbin,150001, China.; 7 School of Life Science and Technology, Harbin Institute of Technology, Harbin, 150001, China.; 8 Drug Engineering and Technology Research Center, Harbin University of Commerce, Harbin, 150001, China.; 9 St John's College William Nicholls Drive, Old St Mellons, Cardiff, CF35YX, United Kingdom.

**Keywords:** Colon adenocarcinoma, KLF7, PDGFB, Sunitinib

## Abstract

Colon adenocarcinoma (COAD) is the most common malignancy of the digestive tract, which is characterized by a dismal prognosis. No effective treatment has been established presently, thus there is an urgent need to understand the mechanisms driving COAD progression in order to develop effective therapeutic approaches and enhance clinical outcomes. In this study, we found that KLF7 is overexpressed in COAD tissues and correlated with clinicopathological features of COAD. Both gain-of-function and loss-of-function experiments have unequivocally demonstrated that overexpression of KLF7 promotes the growth and metastasis of COAD *in vitro* and *in vivo*, while KLF7 knockdown attenuated these effects. Mechanistically, our findings reveal that KLF7 can specifically bind to the promoter region of PDGFB (TGGGTGGAG), thus promoting the transcription of PDGFB and increasing its secretion. Subsequently, secreted PDGFB facilitates the progression of COAD by activating MAPK/ERK, PI3K/AKT, and JAK/STAT3 signaling pathways through PDGFRβ. Additionally, we found that sunitinib can block PDGFB signaling and inhibit COAD progression, offering a promising therapeutic strategy for COAD treatment.

## 1. Introduction

COAD is a frequently occurring malignant tumor found in the gastrointestinal tract. Epidemiological data indicate that COAD incidence and mortality rates are increasing globally^1^. The current treatment modalities for COAD include surgery, chemotherapy, radiotherapy, immunotherapy, and targeted therapy, among others. Due to the insidious onset, most patients are diagnosed in the middle and advanced stages, which limits the effectiveness of surgical treatment^2^. Although scientific advancements have led to the emergence of several effective treatments that can significantly improve patient survival and prognosis, this disease is still characterized by high rates of recurrence, metastasis, and mortality^3^. Therefore, identifying and discovering novel therapeutic targets and drugs is crucial to improving the prognosis of patients with COAD.

Krüppel-like factors (KLFs) are a family of highly conserved zinc-finger transcription factors that play crucial roles in various biological processes in humans, such as regulating gene expression through the binding of targeted gene promoters' GC-rich elements^4^. The KLF proteins are broadly classified into three main groups, with the first group (comprising KLFs 3, 8, and 12) and the third group (comprising KLFs 9, 10, 11, 13, 14, 15, and 16) mainly act as repressors of transcription. In contrast, the second group (including KLFs 1, 2, 4, 5, 6, and 7) plays the role of transcriptional activators^5^. The dysregulation of KLF family proteins has been implicated in various cancers^6^-^8^. The transcriptional activator KLF7 is abnormally expressed in multiple cancers, including pancreatic, endometrial, lung, liver, gastric, breast, and prostate cancers. Numerous studies have demonstrated that KLF7 is crucial in promoting malignant growth and metastasis in multiple tumor types^9^-^14^. It has been reported that KLF7 may contribute to the development and metastasis of pancreatic ductal adenocarcinoma through the stimulation of IFN-driven genes and the preservation of Golgi apparatus integrity^12^. The activation of the Ccdc85c-mediated β-catenin protein pathway by the KLF7/VPS35 axis has been demonstrated to enhance the progression of hepatocellular carcinoma cells^13^. In colorectal cancer, it has been found that transcription factor BHLHE40 can stimulate colorectal tumorigenesis through up-regulating genes like KLF7 and ADAM19^15^. However, the role of KLF7 in COAD is not yet fully understood. Thus, this study examines the expression, function, and molecular mechanism of KLF7 in COAD.

Platelet-derived growth factors (PDGFs) are proangiogenic factors that exert their biological effects in either an autocrine or paracrine manner^16^. It is well-established that PDGF plays a crucial role in tumor angiogenesis, growth, invasion, and migration^17^. PDGFB is a subtype of platelet-derived growth factor that binds to its receptor as a dimeric PDGF-BB, activating the downstream PDGF/PDGFR signaling pathway that can promote the malignant progression of tumors^18^, ^19^. PDGFB, which shares a homologous structure with the simian sarcoma virus oncogene (V-SIS), is a crucial oncogene in COAD^20^. Activation of the PDGF/PDGFR signaling pathway has been shown to promote the growth and metastasis of COAD in various studies. Remarkably, PDGF-BB expression is considerably higher in the serum of COAD patients compared to those with adenoma or healthy controls, thus highlighting its potential as a diagnostic marker for COAD^21^. The transcription factor KLF6 can bind to the PDGFB promoter region and stimulate its expression, ultimately leading to the activation of the mTOR signaling pathway. This activation promotes the growth of renal clear cell carcinoma^22^, ^23^. KLF4, a transcription factor, can also regulate the expression of PDGFB in smooth muscle cells^24^, ^25^. However, the regulatory role of KLF7 in the progression of COAD remains under investigation.

This study demonstrated that KLF7 promotes COAD progression through the PDGFB signaling pathway, while Sunitinib, a potent inhibitor of PDGFR, can hinder this progression.

## 2. Materials and Methods

### 2.1 Cell culture and reagents

The cells were maintained in culture under standard conditions using DMEM medium supplemented with 10% fetal bovine serum (FBS) and 1× P/S solution (100 U/mL penicillin and 100 μg/ mL streptomycin) for LoVo, HCT116, DLD1, and HEK-293T, or RPMI-1640 medium supplemented with 10% FBS and 1× P/S solution for SW620, HT-29, and RKO. Before detecting KLF7 on the PI3K/AKT, JAK/STAT3, and MAPK/ERK signaling pathway, the cells were treated with a conditioned medium containing 0.5% FBS for 24 h. In the sunitinib-treated cells assay, cells treated with sunitinib were assayed in a proliferation assay in a medium containing 2% FBS.

### 2.2 Mice

All animal experiments complied with the guidelines and protocols approved by the Committee on Animal Care at Harbin Medical University. Male athymic BALB/c nude mice, aged 4-5 weeks, were procured from Beijing HFK Bio-Technology Co., Ltd. and housed in a sterile environment. For the xenograft tumor growth assay, 4×10^6^ cells were subcutaneously injected into the flank of mice. After six weeks, the mice were euthanized, and the tumor volume was measured. The formula for calculating tumor volume is Tumor volume (V) = (length × width × width) / 2. In the tail vein injection assay, we used 30-gauge needles to inject 2×10^6^ cells in 100 μL of PBS into the lateral tail vein of mice. After eight weeks, the mice were euthanized, and their lungs were visually inspected for metastases. Additionally, we harvested the tumor for histological examination.

### 2.3 Quantitative reverse transcription-polymerase chain reaction (RT-qPCR)

Total RNA was extracted from cultured cells or frozen colorectal tumor tissues using Trizol (Invitrogen). Total RNA was extracted and reverse-transcribed into cDNA using the ReverTra Ace qPCR RT Kit (Toyobo) following the manufacturer's protocol. Real-time quantitative PCR was performed with the SYBR Premix Ex Taq kit from Takara. The primers utilized in this study are presented in Table S1. To ensure accuracy, we normalized the data to the expression levels of GAPDH.

### 2.4 Western Blotting

The cells were disrupted using a lysis buffer containing 0.5% SDS and 1% protease inhibitor cocktail. The lysates were collected after sonication at 35% amplitude for 30 seconds and boiling for 10 minutes. The protein concentration of the lysates was measured using the bicinchoninic acid (BCA) assay. In this study, roughly 30 μg of protein was loaded onto the gel, followed by the transfer of the proteins onto a 0.45 μm PVDF membrane. The PVDF membrane was blocked using 3% BSA or 5% skim milk and then incubated with the primary antibody overnight at 4°C. Subsequently, the membrane was incubated with the secondary antibody for 1 hour at room temperature. Antibody information can be found in Table S2. Blots were developed using the ECL system (Tanon).

### 2.5 Plasmid and Transfection

To achieve transient overexpression, we separately amplified the full-length cDNA of KLF7 and PDGFB using the primers listed in Table S1 *via* PCR. The amplified cDNA products were cloned into the BamH Ⅰ and Not Ⅰ sites of the pcDNA3.1-3×HA and BamH Ⅰ and Xho Ⅰ sites of the pcDNA3.4(+) plasmids, respectively. To achieve stable gene silencing, the sense and antisense sequences of the shKLF7 oligos (listed in Table S1) were annealed and inserted into the Age I/EcoR I sites of the pLKO.1 vector. For stable gene overexpression, the full-length cDNA of KLF7 was cloned into the Xho Ⅰ/Not Ⅰ sites of the pLVSIN-CMV-puro plasmid vector. To produce the PDGFB promoter Luc plasmid, we inserted the PDGFB promoter sequence into the Kpn Ⅰ/Hind ⅠⅠⅠ site of the pGL4.11 plasmid, which contains the luciferase reporter gene, using the primers listed in Table S1. To generate luciferase plasmids with deletion mutations (R1: AGGGCGGGG, R3: TGGGTGGAG, R6: AGGGGGGGG) in the PDGFB promoter region (-207~+1), we synthesized DNA fragments (211bp) of the PDGFB promoter region with deletions of R1, R3, and R6. These fragments were then amplified by PCR and inserted into the pGL4.11 vector. All plasmid constructs were validated by sequencing. We used the calcium phosphate transfection method for lentivirus packaging to introduce the necessary plasmids into the cells. According to the manufacturer's instructions, the Lipofectamine 3000 reagent (Invitrogen) was used to perform transient transfections of siRNAs (with the siRNA sequences listed in Table S1) or plasmids.

### 2.6 Stable cell lines

To establish stable cell lines, we used lentiviral transduction. This involved transfecting HEK 293T cells with either pLKO.1 or pLVSIN-CMV-puro plasmids and a packaging mix containing VSV-G and Gag-Pol plasmids. After 48 hours of transfection, the lentivirus in the supernatant was collected and filtered, then mixed with polybrene (8 μg/mL) to infect the desired cells. After two days of co-culture with lentivirus, the transduce cells were selected for at least six days using puromycin (2 μg/mL). The effectiveness of overexpression or knockdown was verified by using Western blot analysis or RT-qPCR.

### 2.7 Proliferation assay

After transfecting SW620 and LoVo COAD cells with pcDNA3.1-3×HA-KLF7 or siKLF7 for 48 hours, the cells were seeded at a density of 1,000 cells per well into 96-well plates and cultured for 0, 1, 3, 5, or 7 days. The 3-(4,5-dimethyl thiazolyl-2)-2,5-diphenyltetrazolium bromide (MTT) reagent (5 mg/mL; Sigma) was added to each well, and the cells were incubated for four hours. After incubation, the MTT crystals were dissolved in DMSO and the absorbance was measured at 490 nm using the iMark Microplate Absorbance Reader (Bio-Rad, USA).

To assess cell clones' formation, transfected cells were seeded into 6-well plates at a density of 500 cells per well. The culture medium was changed every five days. After 14 days of culture, the cells were fixed with methanol for one hour, stained with 0.5% crystal violet (Sinopharm Chemical Reagent, China) for one hour, and photographed the colonies using a digital camera. We then counted the number of colonies using Image J software (NIH, Bethesda, MD, USA).

### 2.8 Wound Healing Assay

SW620 and LoVo COAD cells were transfected with pcDNA3.1-3×HA-KLF7 or SiKLF7 for 48 h. The cells were then seeded in a 6-well plate at a density of 3× 10^5^ cells per well and cultured until they reached confluence. Using a 200 μL plastic pipette tip, a scratch was created, and the cells were washed twice with 1×PBS to remove any debris and smooth the edge of the scratch. The medium was subsequently replaced with a serum-free medium. Images were captured immediately after the scratch and 24 and 48 hours later. The images were quantified using Image J software (NIH, Bethesda, MD, USA).

### 2.9 Transwell Assay

Transwell chambers with polycarbonate filters (Corning Cat. No. 3464, 24-well insert, pore size: 8 μm) were used for cell migration assays. The upper section was seeded with 3 × 10^4^ SW620 cells or 1 × 10^4^ LoVo cells in a serum-free medium (150 μL). The lower chamber was filled with medium (500 μL) containing 20% FBS. The plates were subsequently placed in a 37°C incubator for 48 hours. After incubation, non-migrated cells on the upper surface of the chamber were removed using a cotton-tipped swab. The migrated cells on the lower surface of the section were fixed with methanol, stained with 0.5% crystal violet (Sinopharm Chemical Reagent, Shanghai, China), and counted under a microscope. The number of migrated cells was quantified using Image J software (NIH, Bethesda, MD, USA).

### 2.10 ELISA

KLF7 overexpression plasmids, knockdown of KLF7 siRNA, and their respective controls were transiently transfected in SW620 and LoVo cells. After 8 hours of transfection, the culture medium was replaced with a serum-free medium, and the supernatants were collected for testing after an additional 40 hours of incubation. The concentration of PDGF-BB was determined using the Human PDGF-BB Valukine^TM^ ELISA kit (NOVUS, VAL145).

### 2.11 Luciferase assays

We amplified the DNA fragments of the PDGFB promoter (-2000~+1bp) and its truncation bodies using LoVo cell genomic DNA as a PCR template. The amplified fragments were subsequently inserted into the pGL4.11-Basic vector and validated by Sanger sequencing. To investigate the effect of KLF7 on PDGFB promoter activity, SW620 cells were sown in 12-well plates and transfected with pcDNA3.1-3×HA-KLF7 and pGL4.11 luciferase reporter plasmids (both 500 ng/well) along with pGL4.74 control (50 ng/well). After transfecting cells for 48 hours, we collected cell lysates and detected luciferase activities using the dual-luciferase reporter assay system from Promega. The primer sequences utilized in this study are provided in Table S1.

### 2.12 Immunolabeling and histological analysis

The KLF7 (Abcam, ab10802), Ki67 (PTM, 5032), and Vimentin (10366-1-AP) expressions in COAD tissue microarray and mouse tumor tissues were evaluated by immunohistochemical (IHC) staining. Two experienced pathologists independently assessed the staining intensity and extent of staining in the COAD tissue microarray. Digital images of mouse tumor tissues were analyzed with ImageJ software for analysis of KLF7, Ki67, and Vimentin-positive cells. The total percentage positivity for each slide was then calculated and statistically analyzed using GraphPad Prism software.

Mouse tumor tissues were fixed overnight with 4% paraformaldehyde and implanted in paraffin. Paraffin blocks were then cut into 10 µm thick sections. The mouse tissue sections were subjected to standard hematoxylin-eosin (H&E) and immunohistochemical staining protocols. The sections underwent autoclaving with citrate buffer (pH 6.0) for antigen retrieval. Subsequently, endogenous peroxidase activity was slaked with 3% hydrogen peroxide, and the sections were washed with PBS. Nonspecific dressing sites were hindered with 2% bovine serum albumin for 1 hour. The cells were then covered overnight at 4 °C with primary antibodies. The following day, appropriate secondary antibodies were applied and covered for 1 hour at room temperature, followed by detection using a horseradish peroxidase-conjugated secondary antibody. The color reaction of the substrate 3,3'-diaminobenzidine (DAB) was observed under the microscope as a brown color. Meyer Hematoxylin H&E Staining, incubated with 1% HCl, washed, stained with 0.5% eosin, and cleaned again with 100% alcohol before mounting for microscopy observation.

### 2.13 RNA-Seq Analysis

RNA extraction was performed using Trizol high-performance reagents (Invitrogen) to obtain total RNA from the samples. Subsequently, RNA libraries were sequenced using the Illumina Novaseq™ 6000 platform, carried out by LC Bio-Technology CO., Ltd (Hangzhou, China). DESeq2 software was used to analyze differential expression on two groups, each comprising three biological replicates. Genes with a false discovery rate (FDR) lower than 0.05 and absolute fold change of 2 or greater were defined as differentially expressed. An enrichment analysis of KEGG was performed to gain further insights into the biological significance of the differentially expressed genes.

### 2.14 Chromatin Immunoprecipitation Assay (ChIP)

To cross-link the chromatin, the cells were treated with 1% formaldehyde for 10 minutes at standard room temperature. Subsequently, the cells were washed with PBS and lysed, and the genomic DNA was fragmented by sonication into small fragments. The sonicated chromatin was mixed with nuclear lysis buffer until the final SDS concentration reached 0.1%. Before immunoprecipitation, the chromatin was pre-cleared with protein A/G magnetic beads that had been washed for 1 hour at 4 °C with continual rotation with 500 μL of buffer. Immunoprecipitation was performed overnight at 4 °C with an end-to-end process, using specific antibodies such as Anti-HA-Tag antibody (Abmart, M20003S) and Anti-IgG antibody (Beyotime, A7016). To elute the immunoprecipitated DNA from the beads, we incubated it with 200 μL of elution buffer (50 mM Tris-HCl pH 8, 10 mM EDTA, 1% SDS) at 65°C for one hour. The DNA was then purified through phenol extraction and ethanol precipitation. Subsequently, qPCR and PCR were used to amplify the relevant binding sites on the promoters. The primer sequences are shown in Table S1.

### 2.15 Clinical samples

With the patients' entire agreement, we acquired new COAD samples from Harbin Medical University's Fourth Affiliated Hospital. The study was authorized by the Research Ethics Committee of Harbin Medical University's Fourth Hospital. It was carried out in strict accordance with the International Ethical Guidelines for Biomedical Research Involving Human Beings. The cDNA microarray (HColA060CS02) and tissue microarray (HColAde060CS01) of COAD tissues were provided by Shanghai Outdo Biotech Company for this study. The company's Ethics Committee approved the use of these microarrays in research.

### 2.16 Bioinformatics prediction

We obtained expression data for 329 samples, consisting of 288 COAD samples and 41 normal samples, from the TCGA database (https://tcga-data.nci.nih.gov/tcga/). In addition, we retrieved four sets of COAD microarray expression data from the GEO database (https://www.ncbi.nlm.nih.gov/geo/). Using the R package limma, we analyzed the differential expression levels of the KLF gene family between COAD and normal samples. We then conducted patient survival analysis, Pearson correlation analysis, and clinical relevance analysis using GEPIA. Finally, we used the XIANTAO platform (www.xiantaozi.com) to evaluate KLF7 expression in COAD from the TCGA between tumor and matching normal tissues and to examine the connection of KLF7 expression levels with the TNM stage.

### Statistical analysis

Statistical analysis was conducted using GraphPad Prism 8.0.2. Unless specified otherwise, continuous variables were reported as mean ± SD. To compare the two groups, a two-tailed Student's t-test was applied. Multiple comparisons were assessed using two-way ANOVA. The Spearman correlation coefficient was utilized for conducting correlation analysis. If the p-value was not significant (ns), it was denoted as such. A p-value less than 0.05 (*) indicated statistical significance, while p-values less than 0.01 (**) and 0.001 (***) represented higher degrees of significance.

## Results

### 3.1 KLF7 is significantly upregulated in COAD

**Figure 1:**
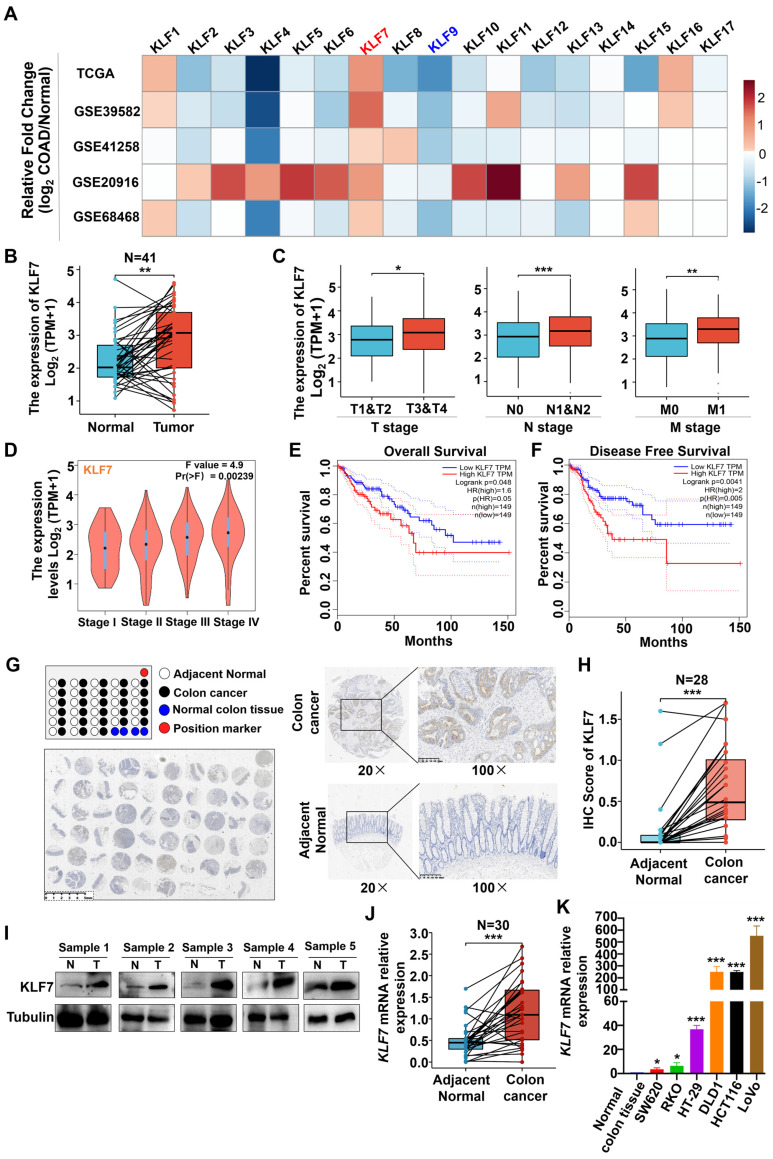
KLF7 is significantly upregulated in COAD. (A) The heatmap displays the fold changes in mRNA expression levels of KLF family members between COAD tumors and normal colon tissues, as obtained from the GEO and TCGA data sets. (B-D) mRNA expression of *KLF7*. The *KLF7* expression in paired COAD and adjacent normal tissues from TCGA (B) and in COAD with different clinical stages from TCGA (C) and GEPIA (D) were analyzed. (E, F) Survival analysis using the GEPIA database. (G) Representative IHC staining images of COAD tissue microarrays. Tumor (up) and normal mucosa (down) from the same patient were presented. (H) IHC staining of KLF7 in the paired COAD samples. (I) KLF7 protein was determined for five-paired COAD samples by Western blot(n=5). (J, K) mRNA expression of KLF7 in paired COAD samples (J) (n=30), as well as three pooled normal colon tissues and COAD cell lines (K) (n=3). Pooled normal colon tissues were used as control. *P< 0.05, **P< 0.01 and ***P< 0.001.

To investigate the potential involvement of KLF family members in the development of COAD, we analyzed the expression levels of KLF1~17 across multiple data sets, including TCGA and GEO (GSE39582, GSE41258, GSE20916, GS68468). Among the *KLF* genes, consistent and significant alterations of *KLF7* and *KLF9* were observed in COAD across all five data sets (Fig. 1A). In the TCGA dataset, the expression of *KLF7* was found to be significantly upregulated (Fig. 1B). In contrast, the expression of *KLF9* was found to be significantly downregulated in COAD tumors as compared to the adjacent non-tumor tissues (Fig. S1). Further analysis revealed that *KLF9* expression levels were not associated with COAD TNM stages or survival rates (Figs. S2-S4). On the contrary, *KLF7* expression was associated with TNM stages, showing higher expression in stage T3 or T4 relative to T1 or T2 of COAD, higher expression in lymph node metastasis positive (N1&N2) or distant organ metastasis-positive (M1) COAD compared to lymph node metastasis negative (N0) COAD or non-distant metastatic (M0) COAD (Fig. 1C). The correlation between *KLF7* expression and COAD stages was also confirmed in the COAD patients from GEPIA (Fig. 1D). Furthermore, higher expression of *KLF7* was associated with worse prognostic outcomes, as demonstrated by lower overall and disease-free survival rates in COAD patients with high *KLF7* expression in comparison to those with low *KLF7* expression (Fig. 1E-F). Therefore, *KLF7* was selected as the target for further validation.

To validate the aforementioned database analysis results, we conducted an immunohistochemical (IHC) assessment of KLF7 protein expression in COAD tissue microarrays. The results indicated that KLF7 protein expression in COAD tumors was higher than in adjacent non-tumor tissues (Fig. 1G-H). Furthermore, western blot analysis of five paired tumor-normal human COAD samples revealed that KLF7 protein expression is upregulated in tumors relative to adjacent normal tissues (Fig. 1I). The upregulation of *KLF7* in COAD tumors was further confirmed through RT-qPCR (Fig. 1J). We also observed that *KLF7* expression is distinctly upregulated in COAD cell lines compared to normal colon tissue (Fig. 1K). These findings suggest that *KLF7* may act as an oncogene in COAD and contribute to disease progression.

### 3.2 KLF7 promotes COAD cell proliferation and migration

**Figure 2:**
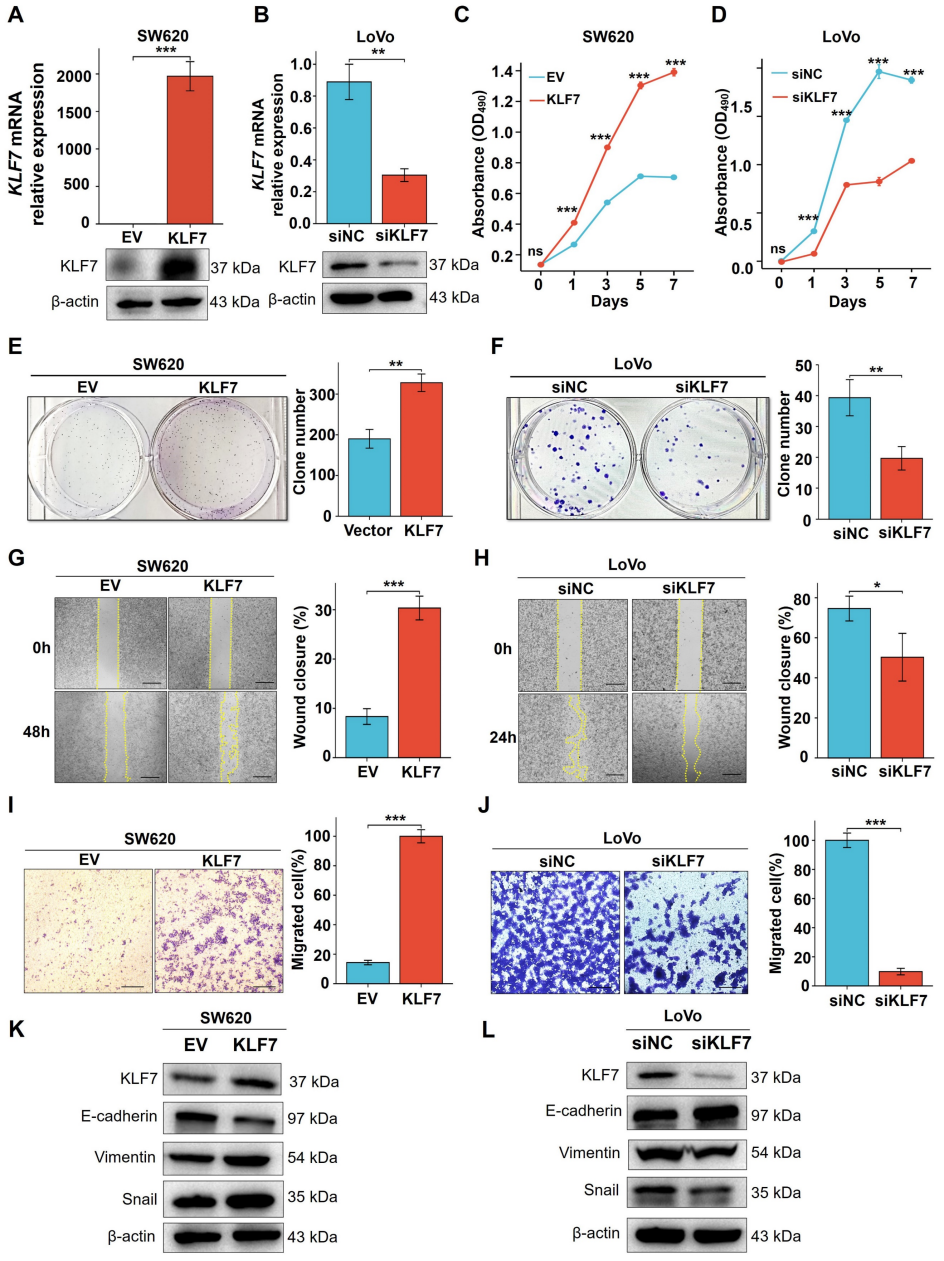
KLF7 promotes the proliferation and migration of COAD cells *in vitro*. (A, B) Real-time PCR and Western blot analysis confirmed the transient upregulation of KLF7 expression in SW620 cells, as well as the transient silencing of KLF7 expression in LoVo cells (n=3). A representative blot was shown. (C, D) MTT assays were used to plot growth curves (n=3). (E, F) Representative images of colony formation assays and corresponding colony counts (n=3). (G, H) Wound-healing assays were performed on the COAD cell lines (n=3). (I, J) Migration assays were performed on the COAD cell lines (n=3). (K, L) Western blot analysis of EMT signaling pathways in indicated COAD cells. Representative blot was shown. Bar, 200 µm (G, H) or 100 µm (I, J). *P< 0.05, **P< 0.01 and ***P< 0.001.

KLF7 has been identified as a critical cell proliferation and migration regulator in multiple cancer types^12^, ^13^, ^26^, ^27^. To investigate the influence of KLF7 on COAD, KLF7 was overexpressed in SW620 cells with low expression or knocked down in the LoVo cells with high expression. Confirmation of the efficiency of transient overexpression or knockdown was carried out through both RT-qPCR and Western blotting (Fig. 2A-B). The MTT assay demonstrated a significant increase in SW620 cell proliferation upon transient KLF7 overexpression compared to the control group (Fig. 2C), while transient KLF7 knockdown notably suppressed LoVo cell proliferation compared to the control group (Fig. 2D). Furthermore, transient overexpression of KLF7 increased the colony-forming capacity of SW620 cells (Fig.2E). In contrast, the knockdown of KLF7 led to a decrease in the colony-forming ability of LoVo cells (Fig. 2F). To assess the impact of KLF7 on cell migration, we performed wound-healing and transwell migration assays. The findings indicated that transient overexpression of KLF7 in SW620 cells accelerated the scratch healing of cells (Fig. 2G), whereas knockdown of KLF7 in LoVo cells decreased this ability (Fig. 2H). Similarly, the transwell migration assay demonstrated that transient KLF7 overexpression increased SW620 cell migration (Fig. 2I), whereas KLF7 knockdown decreased LoVo cell migration (Fig. 2J). Moreover, we investigated the effects of KLF7 on the expression of EMT markers in COAD cells. We found that transient overexpression of KLF7 increased EMT in SW620 cells, shown by decreased expression of E-cadherin and increased expressions of mesenchymal markers Vimentin and Snail (Fig. 2K). Conversely, our study revealed contrasting outcomes in LoVo cells following KLF7 knockdown (Fig. 2L). These above results collectively indicate that KLF7 promotes both the proliferation and migration of COAD cells.

### 3.3 KLF7 knock-down suppresses COAD xenograft growth and lung metastasis

**Figure 3:**
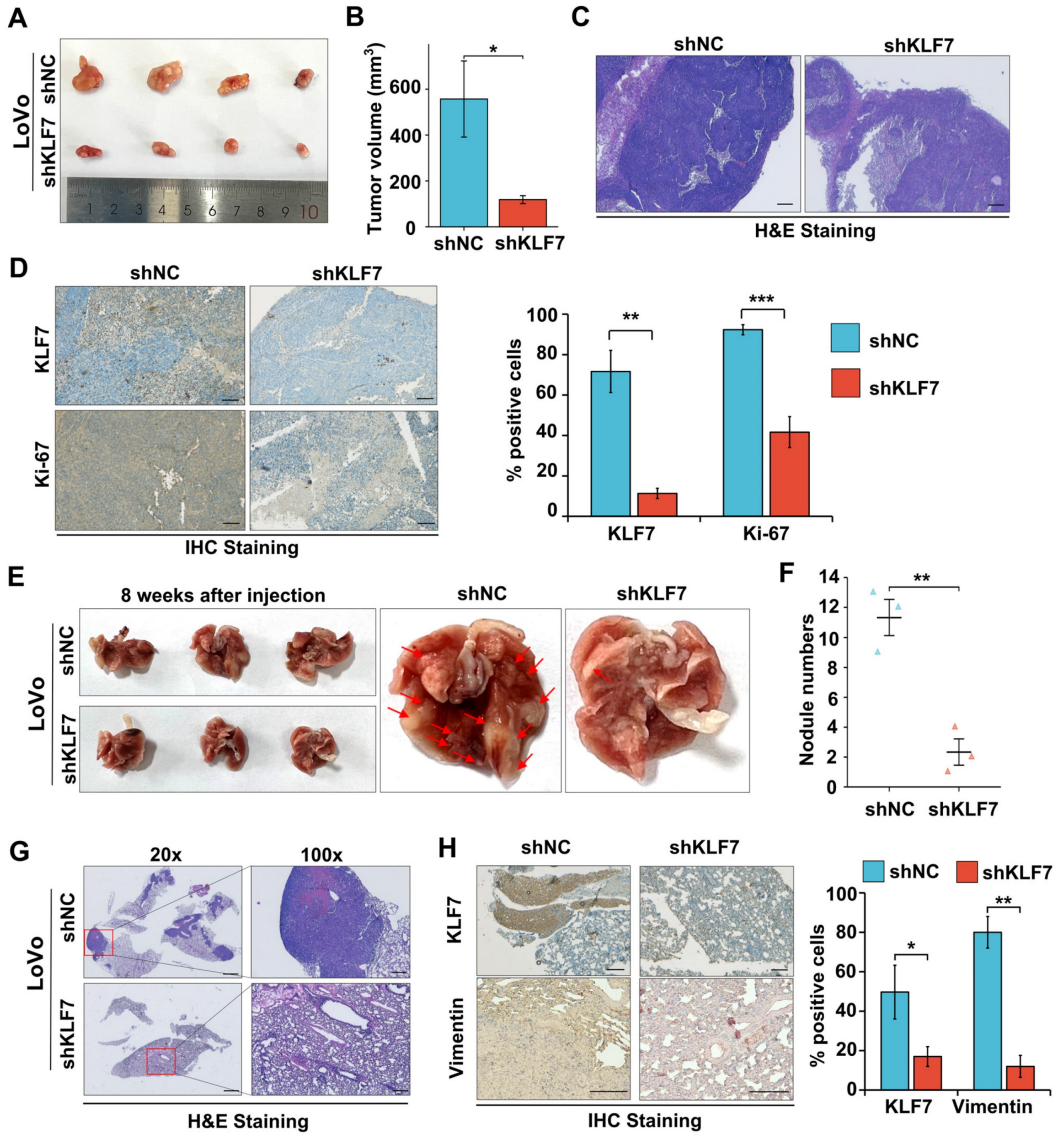
Knockdown of KLF7 inhibits the growth and lung metastasis of COAD xenograft. (A, B) Male BALB/c nude mice were inoculated with the stable KLF7 knockdown and control cells in their right flank. Representative gross images (A) and subcutaneous xenograft tumor volumes (B) in the indicated groups (n = 4). (C, D) Representative staining results from H&E (C) and IHC (D Left) in the subcutaneous xenograft tumor, quantification of IHC staining for KLF7 and Ki67 are also presented (D right) (n = 4). (E, F) Representative gross images (E) and the number of lung metastases (F) from Male BALB/c nude mice that were injected with the stable KLF7 knockdown cells and control cells *via* the tail vein (n = 3). (G, H) Representative staining results from H&E (G) and IHC (H) for KLF7, Vimentin in lungs with metastatic nodules, quantification results of IHC staining are also presented (n = 3). Bar,1000 µm (G-20×), 200 µm (C, G-100×), and 100 µm (D, H). *P < 0.05, **P < 0.01 and ***P < 0.001.

To validate the role of KLF7 in COAD *in vivo*, A stable knockdown or overexpression KLF7 cell line was generated in LoVo or SW620 cells. The efficiency of knockdown or overexpression was assessed by using RT-qPCR (Fig. S5A-B). COAD xenograft was established by injection of Lovo cells with stable KLF7 knockdown (shKLF7) or negative control (shNC) in BALB/c nude mice. The results showed that KLF7 stable knockdown significantly decreased tumor volume compared to the control group (Fig. 3A-B). The H&E staining results indicated a reduction in the density of cancer cells within the tumor in the KLF7 stable knockdown group (Fig. 3C). IHC staining revealed that the cancer cell proliferation was significantly suppressed in the KLF7 stable knockdown group, as indicated by decreased Ki-67 staining (Fig. 3D). Conversely, KLF7 stable overexpression promoted tumor growth, significantly increasing tumor volume and weight (Fig. S5C-F).

To gain deeper insights into the influence of KLF7 on tumor metastasis, we established an experimental metastasis model by intravenously injecting COAD cells into the tail vein of BALB/c nude mice. After eight weeks, we euthanized the mice and assessed the number of pulmonary metastases. Our findings showed that KLF7 stable knockdown led to a decrease in the number of metastatic nodules (Fig. 3E-F), while stable overexpression of KLF7 increased metastasis (Fig. S6A-B). The results of representative H&E and IHC staining revealed that stable knockdown of KLF7 resulted in a decrease in the number of metastatic colonies in the lungs (Fig. 3G-H). Conversely, stable overexpression of KLF7 led to an increase in metastasis (Fig. S6C-D). These *in vivo* results demonstrate that KLF7 promotes COAD xenograft growth and lung metastasis*.*

### 3.4 KLF7 promotes COAD progression by activating the JAK/STAT3, PI3K/AKT, MAPK/ERK signaling pathways

**Figure 4:**
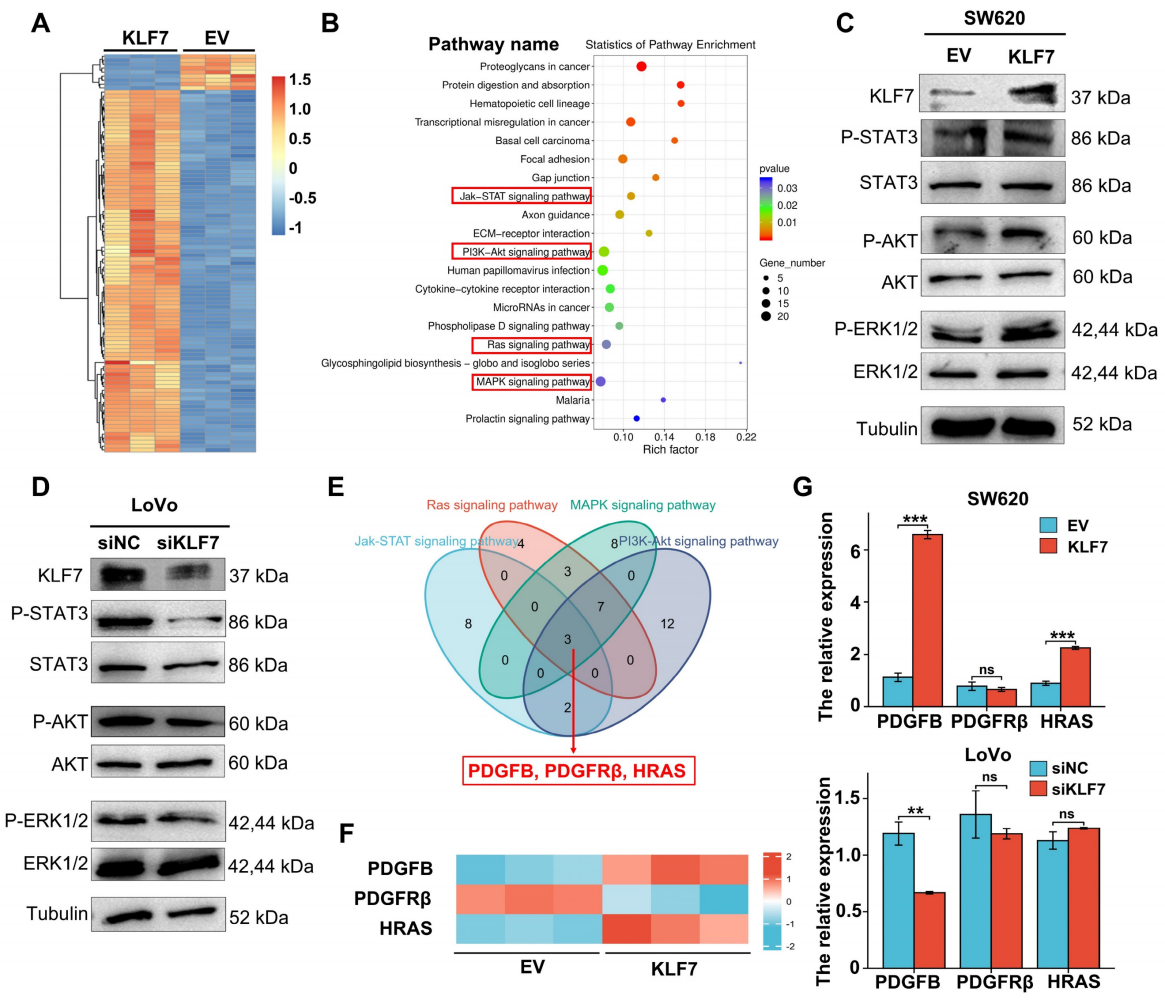
KLF7 promotes activation of JAK/STAT3, PI3K/AKT, MAPK/ERK signaling pathways. (A) A heatmap of the mRNAs of the significant DEGs between empty vector (EV) and KLF7 stable overexpressed cells(n=3). (B) Selection and enrichment significates in KEGG signaling pathways; 20 most important signaling pathways are shown and sorted based on *P* value. (C, D) Representative Western Blot analysis of the activation of enriched MAPK/ERK, JAK/STAT3, and PI3K/AKT pathway in transient vectors (empty vector (EV) or KLF7) transfected SW620 cells (C), or indicated transient siRNA transfected Lovo cells (D) that treated with conditioned media (0.5% FBS). (E) Venn diagrams display the significant DEGs that were similarly altered in the MAPK/ERK, RAS, JAK/STAT3, and PI3K/AKT signaling pathways. (F) Heatmap representing the mRNA expression of *PDGFB*, *PDGFRβ,* and *HRAS* genes between empty vector (EV) andKLF7 transient overexpressed cells(n=3). (G) Effects of KLF7 on the mRNA expression of *PDGFB*, *PDGFRβ,* and *HRAS* in the indicated cells (n=3). ns, not significant, ** P<0.01 and *** *P* <0.001.

To further investigate the downstream signaling pathways of KLF7, we conducted RNA-seq analysis in KLF7 stable overexpression or empty vector (EV) control SW620 cells. Differentially expressed genes (DEG) were selected (|log_2_FC|≥1, *P*<0.05), and their enriched signaling pathways were identified (Fig. 4A, Table S3), showing that KLF7 regulated several signaling pathways associated with cancer, including MAPK/ERK, RAS, JAK/STAT3, and PI3K/AKT (Fig. 4B, Table S4). Western blot confirmed that KLF7affected these enriched pathways, as shown by increased phosphorylation of STAT3, AKT, and ERK in KLF7 transient overexpression cells (Fig. 4C). Conversely, KLF7 transient knockdown decreased the phosphorylation levels of these proteins (Fig. 4D). To identify common DEGs among the four signaling pathway datasets, we employed a Venn diagram, revealing three shared DEGs: PDGFB, PDGFRβ, and HRAS (Fig. 4E). RNA-seq results showed that overexpression of KLF7 in SW620 cells upregulated the expression of PDGFB and HRAS and downregulated the expression of PDGFRβ (Fig. 4F). RT-qPCR analysis confirmed the upregulation of PDGFB and HRAS in SW620 cells with transient KLF7 overexpression and a decrease in PDGFB expression upon KLF7 knockdown in LoVo cells (Fig. 4G). Notably, there was no significant alteration of PDGFRβ and HRAS expression along with transient KLF7 knockdown or overexpression (Fig. 4G). These findings suggest that KLF7 potentially activates the MAPK/ERK, RAS, JAK/STAT3, and PI3K/AKT signaling pathways, possibly mediated by PDGFB.

### 3.5 KLF7 binds to the promoter region of PDGFB and positively regulates the expression of PDGFB

**Figure 5:**
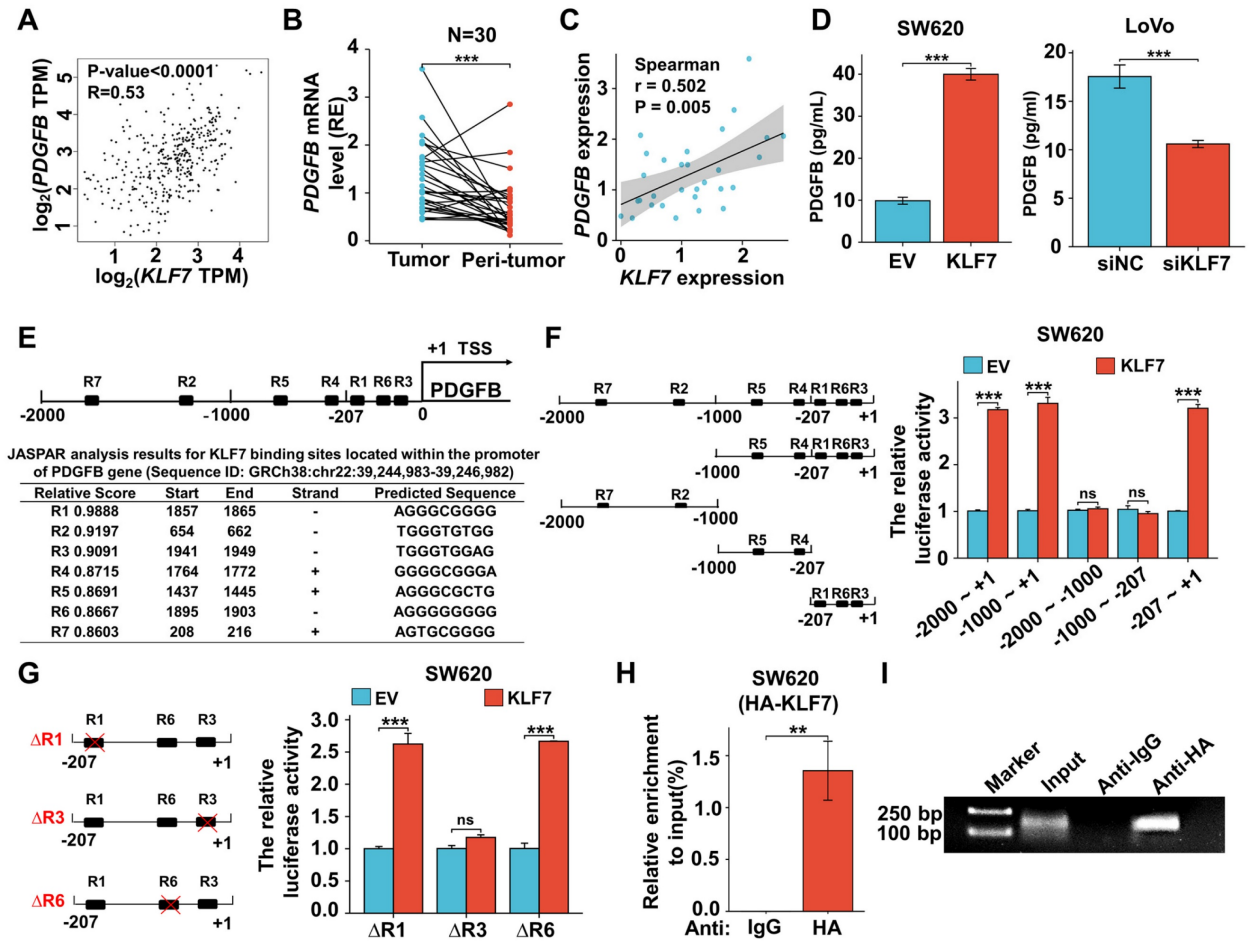
KLF7 positively regulates the expression of PDGFB. (A) Correlation between KLF7 and PDGFB expression in the GEPIA database. (B) *PDGFB* mRNA expression in 30 paired COAD samples (n = 30). (C) Spearson's correlation analysis of *KLF7* and *PDGFB* mRNA expression ratio (tumor/peri-tumor) in COAD patients (n = 30). Spearson's correlation coefficient (r) and *P*-values are shown. (D) ELISA in transient KLF7-overexpressing SW620 cells orKLF7-knockdown LoVo cells cultured at low serum condition (n = 3). (E) Seven KLF7-binding sites on the PDGFB promoter were predicted *via* Jaspar. R indicates the potential binding region. (F) The constructs with different predicted KLF7 were generated (left), and the activity of the PDGFB promoter was determined by a dual-luciferase reporter assay (right) (n = 3). (G) Dual-Luciferase Reporter Assay using PDGFB promoter construct (-207~+1) with R1, R3, or R6 deletion (∆) in SW620 cells (n = 3). (H, I) The ChIP assay was conducted by immunoprecipitating DNA with either anti-IgG or anti-HA antibody in SW620-KLF7 cells, followed by qPCR (H) and PCR(I) amplification using specific primers of PDGFB promoter region -207~+1 (n = 3). ns, not significant, **P < 0.01 and ***P < 0.001.

To clarify the regulatory relationship between KLF7 and PDGFB, we initially conducted an mRNA expression analysis of KLF7 and PDGFB in COAD samples sourced from the TCGA database, utilizing the GEPIA tool. This analysis unveiled a statistically significant and positive correlation between *KLF7* and *PDGFB* expression in COAD (Fig. 5A). Subsequently, we extended our investigation to clinical COAD samples. In these samples, both *KLF7* and *PDGFB* exhibited up-regulation in tumor tissues compared to adjacent normal tissues. (Fig. 5B, Fig.1J), and we observed a positive correlation between *KLF7* and *PDGFB* expression (Fig. 5C). In COAD cell lines, the intracellular protein level of PDGFB was not affected by transient overexpression or knockdown of KLF7 (Fig. S7). However, KLF7 promoted the secretion of PDGFB in COAD cells (Fig. 5D), which was congruent with the observed changes in the intracellular mRNA levels of PDGFB (Fig. 4G). The above results suggest that KLF7 positively regulates the expression of PDGFB in COAD.

As a transcription factor, KLF7 may promote the transcription of PDGFB by binding to the promoter region. To precisely pinpoint the KLF7 binding sites on the PDGFB promoter, we performed JASPAR analysis and identified seven putative KLF7 binding regions (named R1~R7) (Fig. 5E). To confirm the direct transcriptional regulation relationship between KLF7 and PDGFB, we carried out the dual-luciferase assay. Our findings demonstrated that transient KLF7 overexpression significantly increased the transcriptional activity of the PDGFB promoter by binding to the region from -207 to +1 bp (R1,3,6) upstream of the PDGFB transcription start site (Fig. 5F). Deletion of the R3(TGGGTGGAG) effectively counteracted the transcriptional activity of the PDGFB promoter induced by KLF7, indicating that KLF7 directly activates PDGFB transcription through binding to the R3 sequence (Fig. 5G). ChIP assay and PCR amplification also indicated that KLF7 binds to the -207 to +1 bp (R1,3,6) region upstream of the PDGFB transcription start site (Fig. 5H-I). Hence, our findings conclusively establish that KLF7 possesses the ability to bind to the PDGFB promoter region, exerting a positive regulatory influence on its expression.

### 3.6 KLF7 promotes COAD progression by activating JAK/STAT3, PI3K/AKT and MAPK/ERK signaling pathways through PDGFB

**Figure 6:**
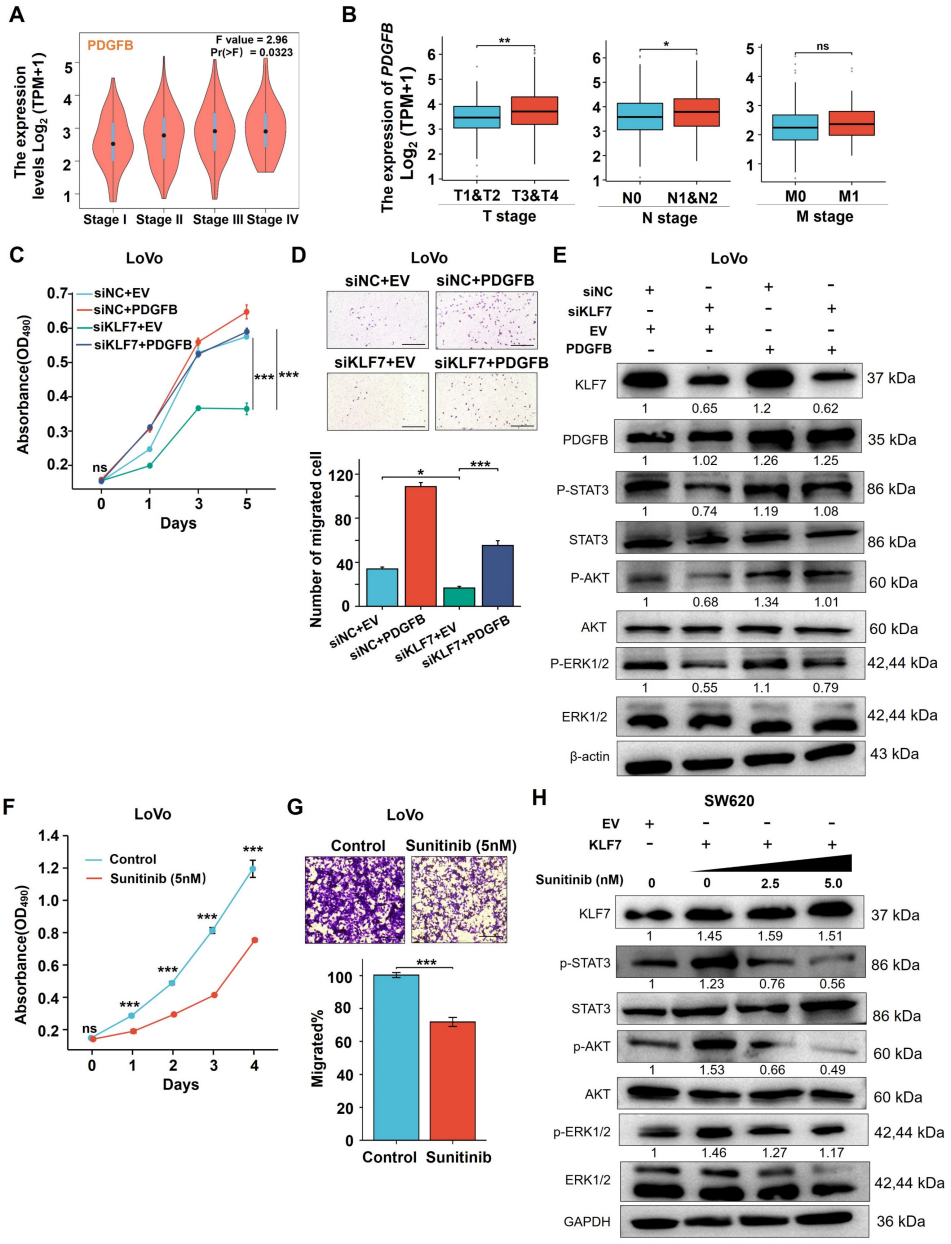
KLF7 promotes COAD progression by activating the MAPK/ERK, PI3K/AKT, and JAK/STAT3 signaling pathways through PDGFB. (A-B) *PDGFB* expression in COAD with different stages in GEPIA (A) and TCGA (B). (C-E) Effects of PDGFB in KLF7 transient knockdown or control LoVo cells were determined by MTT (C), migration (D), and Western Blot analysis (E). Cells were transfected with siRNA negative control (NC) or siKLF7 for 12 hours, followed by an additional 48 hours of transfection with empty vector (EV) or PDGFB overexpression vector (PDGFB). a Thereafter cells were collected for Western blot analysis, or seeded for MTT and Transwell migration assay (n=3). (F, G) MTT assays (F) and Transwell migration assay (G) were used to assess the effects of Sunitinib (5 nM) on LoVo cells (n=3). (H) Western blot analysis of the PDGFB downstream signaling in SW620 cells treated with the indicated concentration of Sunitinib for 48 hours after 12 hours transfection with empty vector (EV) or KLF7 overexpression. Representative images of the Transwell migration assay (D, G) and Western blot (E, H) are presented. Bar, 200 µm. ns, not significant, *P<0.05, ** *P*<0.01 and ***P<0.001.

To explore the regulatory role of PDGFB on the growth of COAD mediated by KLF7, we performed a correlation analysis between *PDGFB* expression and COAD clinical stage in the TCGA database *via* GEPIA. Notably, COAD patients exhibiting elevated *PDGFB* mRNA levels also displayed higher clinical stages, suggesting a positive correlation between *PDGFB* mRNA expression and the clinical stage of COAD patients (Fig. 6A). Additionally, PDGFB expression levels were significantly higher in T3 or T4 stage COAD compared to T1 or T2 stage COAD. Likewise, PDGFB expression exhibited a significant increase in metastatic lymph node-positive (N1&N2) COAD compared to metastatic lymph node-negative (N0) COAD. However, there were no significant differences in PDGFB expression levels between cases of distant organ metastasis (M1) and those without distant organ metastasis (M0) COAD (Fig. 6B). To further assess the impact of restoring PDGFB expression on the effects of KLF7 knockdown in COAD cell proliferation and migration, we conducted MTT and Transwell assays. The results demonstrated a marked reduction in the proliferation and migration capacities of COAD cells in the siKLF7+EV group compared to the siNC+EV group. Notably, in the siKLF7+PDGFB group, there was a significant increase in the proliferation and migration abilities of COAD cells compared to the siKLF7+EV group (Fig. 6C-D). These findings indicate that the restoration of PDGFB expression levels enhances the proliferation and migration capabilities of COAD cells with KLF7 knockdown.

Furthermore, we explored whether restoring the expression level of PDGFB could affect the regulatory effect of KLF7 knockdown in the MAPK/ERK, PI3K/AKT, and JAK/STAT3 signaling pathways. To assess this, we quantified the expression of core proteins of these pathways by Western blotting. The results revealed that the phosphorylation levels of STAT3, AKT, and ERK proteins were down-regulated in the siKLF7+EV group compared to the siNC+EV group, whereas there was a significant upregulation in the phosphorylation levels of STAT3, AKT, and ERK proteins in the siKLF7+PDGFB group compared to the siKLF7+EV group (Fig. 6E). These findings strongly suggest that PDGFB represents one of the downstream targets of KLF7 in regulating MAPK/ERK, PI3K/AKT, and JAK/STAT3 signaling pathways in COAD. Consequently, blocking the PDGFB signaling pathway holds promise for restraining the progression of COAD characterized by KLF7 overexpression.

Sunitinib, a small molecule inhibitor of PDGFRβ, effectively hinders the activation of downstream kinases by binding to PDGFBB to PDGFRβ^28^. We assessed the impact of sunitinib on the proliferation and migration abilities of COAD cells overexpressing KLF7 through MTT and Transwell assays, revealing that sunitinib could suppress the proliferation and migration capabilities of COAD cells with transient KLF7 overexpression (Fig. 6F-G). To prove whether sunitinib modulates the downstream signaling pathways regulated by KLF7, COAD cells transiently overexpressing KLF7 were exposed to varying concentrations of sunitinib. KLF7 overexpression led to an increase in the phosphorylation of STAT3, AKT, and ERK, which was dose-dependently attenuated by treatment with sunitinib (Fig. 6H). This suggests that sunitinib can impede the activation of downstream pathways governed by KLF7 by obstructing the PDGFB signaling pathway.

Collectively, our findings propose that KLF7 primarily promotes COAD progression by activating the MAPK/ERK, PI3K/AKT, and JAK/STAT3 signaling pathways through PDGFB. Moreover, sunitinib effectively inhibits the proliferation and migration of COAD cells overexpressing KLF7.

## 4. Discussion

Currently, developing an effective therapeutic target for COAD remains a significant challenge. Thus, an exhaustive understanding of the processes underlying COAD progression is essential for developing novel and effective therapeutics. Our study has revealed that the transcription factor KLF7 was upregulated in COAD and promoted its growth and metastasis through the PDGFB/PDGFRβ signaling axis. Sunitinib, a small molecule inhibitor of PDGFRβ, could block the KLF7/PDGFB/PDGFRβ signaling pathway and effectively inhibit the growth of COAD. These findings suggest that targeting the KLF7/PDGFB/PDGFRβ signaling axis may represent a promising therapeutic strategy for COAD.

KLF7, a ubiquitous Krüppel-like factor expressed at low levels in many human tissues^29^, has been found to be overexpressed in various malignancies and participate in processes associated with tumorigenesis and metastasis^30^-^32^. KLF7 protein expression has been reported to be a distinct indicator of poor prognosis in multiple kinds of cancer, including gastric, lung, acute lymphoblastic, and ovarian cancer^26^, ^33^-^35^. Several studies have found that KLF7 enhances tumor growth and metastasis in various cancers^12^, ^13^, ^26^, ^27^. Consistent with previous research, our study found that KLF7 was overexpressed in COAD. Patients with COAD with high KLF7 expression levels exhibited a worse overall survival rate than those with low KLF7 expression levels. Through gain- and loss-of-function experiments, our findings indicate that KLF7 is a critical factor in the initiation and progression of COAD.

Although the mechanism of KLF7 in various digestive tract tumors has been reported, its exact mechanism in the pathogenesis and progression of COAD is not fully understood. KLF7 has many functions in different diseases, each with distinct molecular mechanisms. It regulates signaling pathways, including β-catenin, NF-κB, AKT, STAT3, and TGFβ 13, 36-39. Our RNA-seq data revealed that KLF7 is linked with several cancer signaling pathways, such as PI3K/AKT, JAK/STAT3, and MAPK/ERK in COAD. The PI3K/AKT, JAK/STAT3, and MAPK/ERK signaling pathways are frequently activated in COAD and involved in metastasis^40^-^42^.The PDGF signaling activates the JAK/STAT3, PI3K/AKT, and MAPK/ERK pathways^43^-^45^. Our RNA-seq data indicated that PDGFB was a differentially expressed gene commonly associated with the MAPK/ERK, JAK/STAT3, and PI3K/AKT signaling pathways in COAD cells. Our results demonstrate that PDGFB is a downstream target gene of KLF7 that regulates the signaling mentioned above pathways in COAD cells. A significant positive correlation between KLF7 and the expression level of PDGFB was found in COAD tissues. Moreover, KLF7 promoted the expression of PDGFB intracellularly and extracellularly in COAD cells. KLF7 is classified as a member of the zinc finger family of transcription factors, which can bind to the CACCC element within promoter regions to promote the transcription of downstream target genes^5^. In this study, we revealed that the R3 region (TGGGTGGAG), located at -60 bp to -53 bp upstream of the PDGFB transcription start site, is responsible for KLF7 binding. In this study, we identified that KLF7 functions as a direct regulator of PDGFB. As a transcription factor, KLF7 can bind to the PDGFB promoter, enhancing the transcription and secretion of PDGFB. The secreted PDGF-BB activates the JAK/STAT3, PI3K/AKT, and MAPK/ERK signaling pathways in COAD cells *via* PDGFRβ.

The ability of cancerous cells to proliferate indefinitely is a crucial hallmark of cancer, which is achieved through their ability to simulate external growth signals in an autocrine manner^46^. In COAD cells, the secretion of PDGF growth factors, which are dimeric and bind to PDGFR on the surface of COAD cells, contributes to the progression of COAD^47^. Our results showed that PDGFB is a major downstream target gene of KLF7 and promotes COAD progression. Overexpression of PDGFB restored the proliferation and migration abilities of the COAD cells inhibited by KLF7 knockdown. PDGFB overexpression abolished the inactivation of JAK/STAT3, PI3K/AKT, and MAPK/ERK signaling pathways caused by KLF7 knockdown. Our results suggest a functional relationship between KLF7 and PDGFB during the growth and migration, and targeting this pathway could be a potential therapeutic strategy for COAD treatment.

The use of transcription factors as drug targets has been challenging due to their complex functions and regulatory networks^48^, ^49^. Hence, targeting KLF7 directly cannot be considered a viable drug target. However, targeting downstream effectors or interacting molecules of KLF7, such as PDGFB, may be an effective therapeutic approach to treat COAD. This is because PDGFB signaling is known to promote COAD progression through KLF7. Therefore, targeting the PDGFB/PDGFRβ signaling pathway can effectively alleviate the malignant phenotype of COAD with high KLF7 expression. The PDGFRβ inhibitor, sunitinib, is commonly used to inhibit this pathway^50^. Sunitinib is a multitargeted tyrosine kinase inhibitor that potently inhibits PDGFRβ and is approved for treating advanced kidney cancer, gastric mesenchymal tumors, and advanced pancreatic endocrine tumors^51^, ^52^. Our study found that sunitinib effectively abolished the overexpression of KLF7-activated JAK/STAT3, MAPK/ERK, and PI3K/AKT signaling pathways. Moreover, as the concentration of sunitinib increased, the phosphorylation level of core proteins related to these signaling pathways decreased. This study demonstrates that sunitinib can effectively inhibit the proliferation and migration ability of COAD cells overexpressing KLF7.

**Figure 7:**
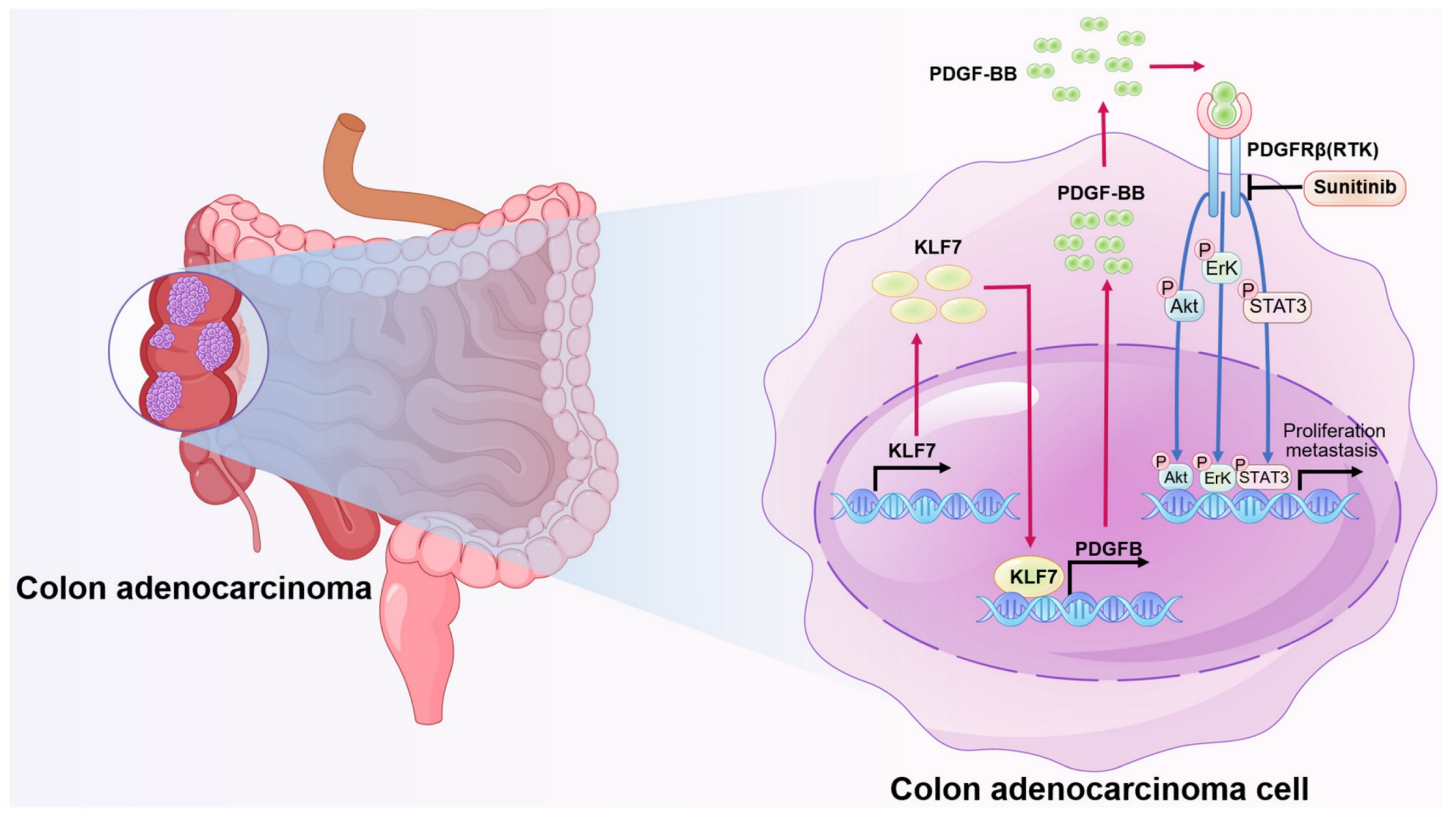
Schematic model illustrating KLF7 promoting COAD progression. KLF7 is overexpressed in COAD and can bind to the promoter region of PDGFB to promote its secretion. The secreted PDGFB then binds to its receptor PDGFRβ, activating downstream pathways that promote COAD progression. Sunitinib can inhibit PDGFRβ, thereby blocking this secretory signaling pathway and inhibiting COAD progression.

In conclusion, this study highlights the critical role of the KLF7/PDGFB/PDGFRβ signaling pathway in the growth and metastasis of COAD cells (Fig. 7). We also identified KLF7 as a key proto-oncogenic transcription factor that regulates PDGFB expression in COAD cells. Importantly, our findings suggest sunitinib may be clinically beneficial for COAD patients overexpressing KLF7.

## Supplementary Material

Supplementary figures and tables.
