# Supplementary material for: KLF7 promotes colon adenocarcinoma progression through the PDGFB signaling pathway
Source: Int J Biol Sci. 2024 Jan 1;20(1):387–402. doi: 10.7150/ijbs.86385 (PMC10750276; doi:10.7150/ijbs.86385)
Supplement: Supplementary file 1 — Supplementary figures and tables. [file ijbsv20p0387s1.pdf]

Supplemental Information for

KLF7 promotes colon adenocarcinoma progression through PDGFB autocrine signaling

Zhicheng Zhang et al.

This file includes:

<u>Figures S1-S6 and legends</u>

- ✓ Figure S1. Expression levels of *KLF9* in COAD in the TCGA database.
- ✓ Figure S2. Correlation between *KLF9* expression level and TNM stage of COAD in the TCGA database.
- ✓ Figure S3. Correlation between *KLF9* expression level and clinical stage of COAD in the GEPIA database.
- ✓ Figure S4. Correlation between *KLF9* expression levels and COAD survival analysis in the GEPIA database.
- ✓ Figure S5. In vivo experiments on the effect of overexpression of KLF7 on tumor growth.
- ✓ Figure S6. In vivo experiments on the effect of overexpression of KLF7 on tumor metastasis.
- ✓ Figure S7. Effect of KLF7 on intracellular PDGFB protein expression levels.

<u>Supplementary Tables S1-S4</u>

- ✓ Table S1. Oligos used for PCR and vector construction
- ✓ Table S2. Antibodies of experiments
- ✓ Table S3. 1143 DEGs by RNA-seq (KLF7 overexpression)
- ✓ Table S4. Kyoto Encyclopedia of Genes and Genomes (KEGG) analysis

Supplementary Figures

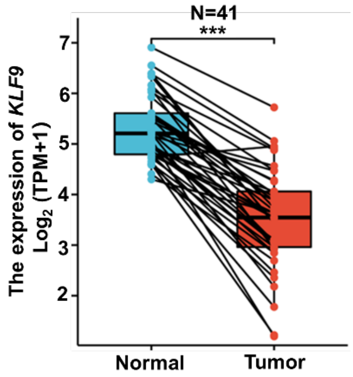

Figure S1. mRNA expression of *KLF9* in paired cancer and para-cancerous normal tissues of patients with COAD patients from TCGA data sets (n=41). ***P<0.001

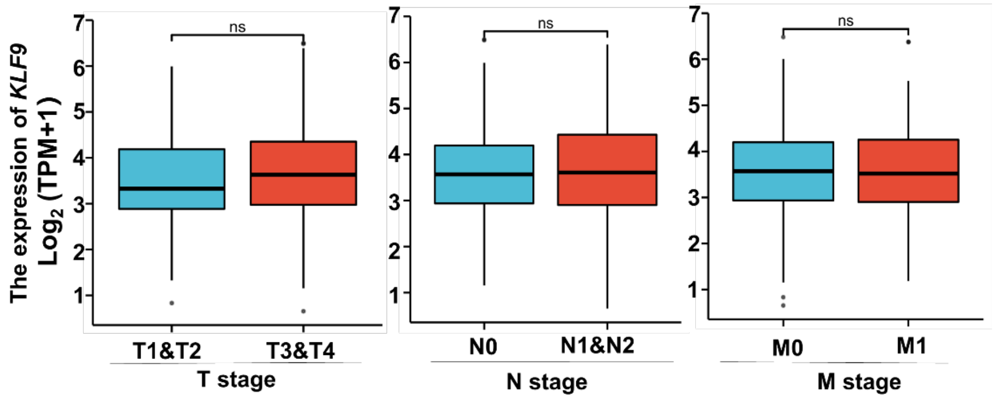

Figure S2. mRNA expression of *KLF9* in different TNM stage of colon cancer in TCGA data sets. ns, *P*>0.05,

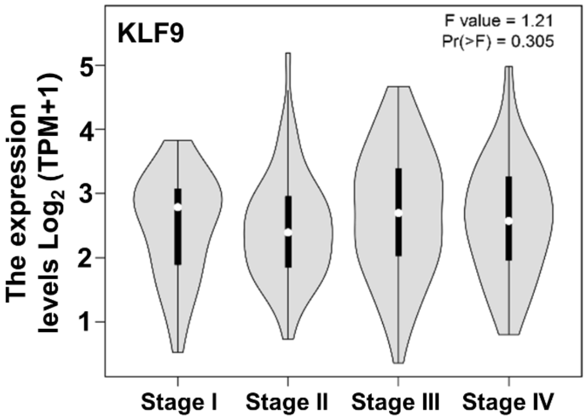

Figure S3. Association between *KLF9* expression and clinical stage of colon cancer in GEPIA database.

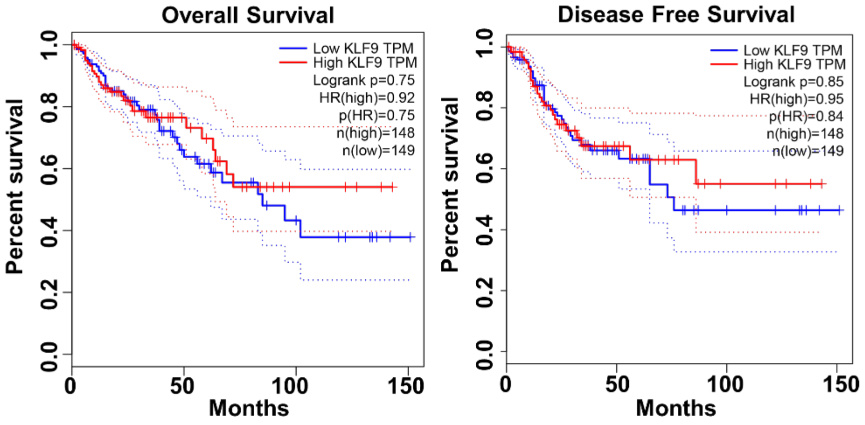

Figure S4. Survival analysis of *KLF9* gene expression using the GEPIA database.

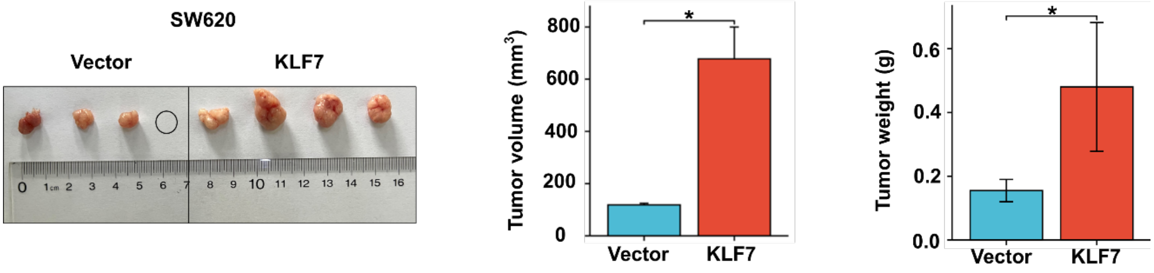

Figure S5. Representative gross view and Subcutaneous xenograft tumor volumes and weights were measured for the different groups. * *P* <0.05

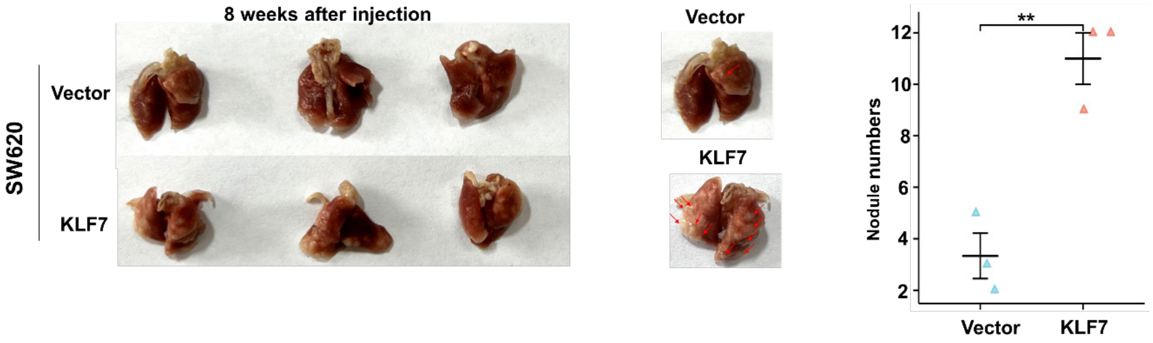

Figure S6. Representative gross view and the amount of the pulmonary metastatic lesions was counted. ** *P* <0.01

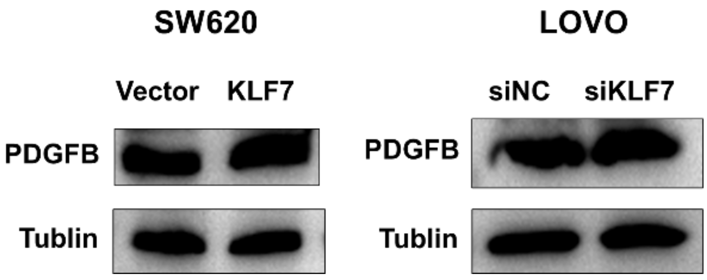

Figure S7. WB analysis of PDGFB protein levels in KLF7-overexpressing SW620 cells and KLF7-knockdown LOVO cells.

Supplementary Tables

Table S1. Oligos used for PCR and vector construction

Oligonucleotide	Sequence (5'-3')
**siRNA**	
si-NC (negative control)	Sense: UUUUCCGAACGUGUCACGUTT
	Antisense: ACGUGACACGUUCGGAGAATT
si-KLF7	Sense: UCAGAUAGCAACUUGUCCCTT
	Antisense: GGGACAAGUUGCUAUCUGA
**shRNAs construction oligos**	
pLKO.1-CTL-shRNA	Sense:
	CCGGTTCTCCGAACGTGTCACGTCTCGAGACGTGACACGTTCGG AGAATTTTTG
	Antisense:
	AATTCAAAAATTCTCCGAACGTGTCACGTCTCGAGACGTGACACGTT CGGAGAA
pLKO.1-KLF7-shRNA	Sense:
	CCGGTCAGATAGCAACTTGTCCCCTCGAGGGGACAAGTTGCTATCTG ATTTTTG
	Antisense:
	AATTCAAAAATCAGATAGCAACTTGTCCCCTCGAGGGGACAAGTTGC TATCTGA
**Plasmids construction**	
pcDNA3.1-3×HA-KLF7	F:TCGAGGATCCATGGACGTGTTGGCTAGTT
	R:TCGAGCGGCCGCTTAGATATGTCTCTTCATGTGGA
pcDNA3.4(+)-PDGFB	F: AAAGGATCCTGAATCGCTGCTGGG
	R: AAACTCGAGCTAGGCTCCAAGGGTC
PDGFB promoter(-2000 ~ +1)	F: AAAGGTACCCCGGTGGAAGAGGGTTTTTA
	R: AAAAAGCTTCCCTTTCAGCTGTTCCGGCC
PDGFB promoter (-1000 ~ +1)	F: AAAGGTACCAGGGCGGATAACAGAGACA
	R: AAAAAGCTTCCCTTTCAGCTGTTCCGGCC
PDGFB promoter (-207 ~ +1)	F: AAAGGTACCTTTTTTCCCCAGGGCCAGAT
	R: AAAAAGCTTCCCTTTCAGCTGTTCCGGCC
PDGFB promoter (-2000 ~ -1000)	F: AAAGGTACCCCGGTGGAAGAGGGTTTTTA
	R: AAAAAGCTTTGTCTCTGTTATCCGCCC
PDGFB promoter (-1000 ~ -208)	F: AAAGGTACCAGGGCGGATAACAGAGACA
	R: AAAAAGCTTGAACTAGGGGTGACCCACC
pLVSIN-KLF7	F:AAAGCGGCCATGGACGTGTTGGCTAGTT
	R:AAAGGATCCGCTTAGATATGTCTCTTCATGTGGA
**RT-qPCR primers**	
KLF7	F: ACAAACAAACAGCCCCAACT

	R: GGTAGCGTTCCAATTCAAGG
PDGFB	F: CTCGATCCGCTCCTTTGATGA
	R: CGTTGGTGCGGTCTATGAG
PDGFRB	F: AGCACCTTCGTTCTGACCTG
	R: TATTCTCCCGTGTCTAGCCCA
HRAS	F: ATGACGGAATATAAGCTGGTGGT
	R: GGCACGTCTCCCCATCAATG
GAPDH	F: TGCACCACCAACTGCTTAGC
	R: GGCATGGACTGTGGTCATGAG
**ChIP-PCR primers**	
PDGFB promoter R136	F: GAAGGGTTGCTCGGCTCTC
	R: CTTTCAGCTGTTCCGGCCT

Table S2. Antibodies and other chemicals used in the experiments

REAGENT or RESOURCE	SOURCE	DENTIFIER
Anti-GAPDH antibody	Proteintch	Cat.# 6004-1-Ig
Anti-actin antibody	Proteintch	Cat.# 60008-1-Ig
Anti-Tublin antibody	Proteintch	Cat.# 66031-1-Ig
Anti-KLF7 antibody	Abcam	Cat.# ab197690
Anti-KLF7 antibody	Santa Cruz	Cat.# sc-398576
Anti-PDGFB antibody	Santa Cruz	Cat.# sc-365805
Anti-HA-Tag antibody	Abmart	Cat.# M20003S
Anti- Phospho-p44/42 antibody	Cell Signaling Technology	Cat.# 9101
Anti- P44/42 antibody	Cell Signaling Technology	Cat.# 4695
Anti- Phospho-Akt antibody	Cell Signaling Technology	Cat.# 4060
Anti- Akt antibody	Cell Signaling Technology	Cat.# 4691
Anti- Phospho -stat3 antibody	Cell Signaling Technology	Cat.# 9145
Anti- Stat3 antibody	Cell Signaling Technology	Cat.# 9139
Anti-IgG antibody(rabbit)	Beyotime	Cat.#A7016
Anti-IgG antibody(mouse)	Beyotime	Cat.#A7028
Anti-Ki67 antibody	PTM biolabs	Cat.#PTM5032

Table S3. 1143 DEGs by RNA-seq in KLF7 overexpressed SW620 cells

Gene	Log_2 Fold Change (KLF7 *vs* Vector)	Regulation
BLOC1S5-TXNDC5	16.08	up
AC090527	15.15	up
AC093525	14.83	up
AL772307	13.99	up
AC126755	13.85	up
HSPE1-MOB4	12.76	up

AL355385	12.38	up
MORC2-AS1	12.23	up
MAFA-AS1	11.83	up
OCLNP1	11.68	up
NBPF19	11.67	up
INMT-MINDY4	11.10	up
NBEAP3	10.98	up
AC022414	10.44	up
AC013271	10.20	up
AC005523	10.11	up
BIVM-ERCC5	9.90	up
POC1B-GALNT4	9.87	up
SFTA1P	9.78	up
Z83844	9.76	up
PAX2	9.67	up
LINC01615	9.55	up
AC232271	9.38	up
AC010343	9.22	up
AC006372	9.21	up
PCDHAC1	9.09	up
KLF7	8.59	up
AC008878	8.04	up
KCNB1	6.67	up
AC004771	6.65	up
COL5A3	5.73	up
FAM25A	5.19	up
UPK3BL1	5.06	up
FXYD4	5.01	up
AL354740	4.90	up
PRPH	4.87	up
CPEB2	4.69	up
AC087632	4.42	up
WNT9A	4.39	up
AC048338	4.29	up
ZBED2	3.95	up
GPR161	3.67	up
KCNE5	3.65	up
KIF26A	3.56	up
PRRX1	3.50	up
ESAM	3.47	up
ZDHHC8P1	3.45	up
DNM1P51	3.43	up
CRIP2	3.40	up
EHD2	3.33	up

AC093838	3.28	up
MMP3	3.21	up
CRIP1	3.20	up
SYNGR3	3.19	up
CDHR5	3.19	up
ROBO4	3.14	up
NACAD	3.11	up
AL049844	2.99	up
HES2	2.98	up
RPLP0P2	2.97	up
TFAP2E	2.93	up
CASC19	2.91	up
CBLL1	2.88	up
CGB5	2.85	up
LRRC3	2.84	up
RNF223	2.80	up
A4GALT	2.74	up
KRT16	2.71	up
TLCD4-RWDD3	2.69	up
INKA1	2.67	up
MATN3	2.65	up
AHNAK2	2.64	up
NCF2	2.59	up
NTN4	2.58	up
LTK	2.57	up
KLC3	2.56	up
AC004233	2.52	up
SLFN5	2.52	up
TTBK1	2.51	up
NPHS1	2.51	up
TUBA1A	2.51	up
MGLL	2.48	up
AVPR2	2.45	up
SCEL	2.44	up
SUSD2	2.43	up
METRNL	2.43	up
GGT1	2.42	up
RRAD	2.42	up
AC091057	2.42	up
AHNAK	2.31	up
FGFBP1	2.22	up
DKK3	2.22	up
SCN9A	2.21	up
TMEM156	2.18	up

KLK10	2.18	up
TUBB2A	2.18	up
RCAN3	2.17	up
ZNF185	2.16	up
RGS11	2.16	up
BOK	2.16	up
MIR22HG	2.15	up
CORO2B	2.14	up
KRT17	2.14	up
KCNK7	2.13	up
SPRR2D	2.11	up
C1QL4	2.11	up
CCDC68	2.11	up
PDLIM2	2.10	up
IL11	2.10	up
RAB3B	2.10	up
RASGEF1A	2.10	up
SYNC	2.05	up
RAB6D	2.04	up
IGFBP1	2.03	up
CDK5R2	2.00	up
CLIC3	1.99	up
AL162258	1.98	up
TRIM7	1.97	up
EIF4E3	1.95	up
HBA1	1.95	up
DENND3	1.93	up
KIF21B	1.92	up
ZFP28	1.90	up
LINC02009	1.89	up
HOXC12	1.89	up
THSD1	1.89	up
TUBB3	1.89	up
GBP3	1.89	up
RTN4R	1.87	up
ZNF600	1.87	up
SDCBP2	1.87	up
SLPI	1.84	up
KL	1.84	up
CDC42EP2	1.84	up
MYADM	1.83	up
CDKL2	1.83	up
IRF9	1.83	up
ARC	1.82	up

TMEM125	1.82	up
ZNF490	1.81	up
KRT81	1.81	up
AL109976	1.81	up
TSPAN1	1.80	up
ALS2CL	1.78	up
MAFA	1.77	up
MACC1	1.76	up
TYMP	1.75	up
FAM214B	1.75	up
CORO2A	1.75	up
CHRFAM7A	1.75	up
NAGS	1.75	up
KCNN4	1.75	up
YWHAH	1.74	up
SULT2B1	1.72	up
AOX1	1.71	up
RTN2	1.71	up
PMP22	1.70	up
FAXDC2	1.70	up
SFN	1.69	up
C6orf132	1.68	up
SLC25A43	1.68	up
EPDR1	1.68	up
MVP	1.67	up
FAM177A1	1.67	up
DLK2	1.67	up
KRT6A	1.66	up
B3GNT7	1.66	up
CAVIN1	1.66	up
DERPC	1.66	up
TPPP	1.65	up
MEAK7	1.65	up
PALM3	1.64	up
FNDC11	1.64	up
KIFC2	1.63	up
TTC9	1.62	up
MAPK11	1.60	up
B3GNT8	1.60	up
RRAS	1.59	up
OSCAR	1.59	up
LYL1	1.59	up
CDKN2D	1.59	up
COX6B2	1.59	up

SPON2	1.59	up
PRSS23	1.58	up
SLC16A4	1.58	up
STYK1	1.57	up
C3orf52	1.57	up
ACP7	1.57	up
DBNDD1	1.57	up
BCL3	1.57	up
HGSNAT	1.56	up
EMP1	1.55	up
RRAGC	1.55	up
SLC4A11	1.55	up
EPHA10	1.54	up
LINC02361	1.54	up
ARHGEF10	1.53	up
MRGPRF	1.53	up
STBD1	1.52	up
PLAU	1.52	up
DUSP18	1.52	up
CD55	1.52	up
N4BP3	1.51	up
UNC5B	1.50	up
THBD	1.50	up
AC092535	1.49	up
MAP1B	1.49	up
CEACAM6	1.49	up
TGFB1I1	1.49	up
MN1	1.49	up
GLIS2	1.48	up
RAET1L	1.48	up
JAZF1	1.48	up
EMP3	1.48	up
AL391422	1.47	up
CACNB1	1.47	up
TDRD7	1.46	up
IL32	1.45	up
LINC01517	1.45	up
RNASE4	1.44	up
STAT5A	1.44	up
RIPK3	1.44	up
C1orf54	1.44	up
DDX58	1.43	up
GJB3	1.43	up
ABCA3	1.43	up

CTDSP1	1.43	up
PKIB	1.42	up
PRSS12	1.41	up
SLC66A3	1.41	up
AC010457	1.40	up
MEX3D	1.40	up
VWA7	1.40	up
TNFSF12	1.40	up
TM4SF5	1.40	up
BHLHA15	1.39	up
WNT5B	1.38	up
FBXL16	1.38	up
TUBB6	1.38	up
TUBB2B	1.38	up
SCN1B	1.38	up
SOCS2	1.38	up
SOCS3	1.38	up
LPAR5	1.37	up
PLXNA3	1.37	up
PLEKHG4B	1.37	up
GPR1	1.36	up
TRIM21	1.36	up
APOL2	1.35	up
CEACAM5	1.35	up
ACY3	1.35	up
AC108488	1.35	up
NDUFC2-KCTD14	1.35	up
L1CAM	1.34	up
STX1A	1.34	up
ATL3	1.34	up
RHOF	1.33	up
KCTD21	1.33	up
TLCD2	1.33	up
GAS2L1	1.33	up
PGM2L1	1.33	up
PTPN21	1.32	up
FSTL3	1.32	up
PLD2	1.32	up
TRPM4	1.31	up
NYAP1	1.31	up
SLC10A3	1.31	up
ITGA3	1.30	up
RASSF8	1.30	up
RGCC	1.30	up

RHOC	1.30	up
TCEA1	1.30	up
TEN1	1.30	up
STX2	1.29	up
SBSPON	1.29	up
SORBS3	1.29	up
PDGFB	1.29	up
SRPK3	1.29	up
CAMK2N1	1.29	up
OPTN	1.28	up
CAPG	1.28	up
IL18	1.28	up
PHLDA3	1.28	up
PCDH1	1.28	up
IRF7	1.28	up
CLIC5	1.27	up
HSF2BP	1.27	up
TPRG1L	1.27	up
FLNB	1.26	up
INPP5E	1.26	up
PLEC	1.26	up
TRIM36	1.26	up
LMTK3	1.25	up
FABP3	1.25	up
ROCK1P1	1.24	up
NOTCH1	1.24	up
PLA2G15	1.24	up
JUP	1.24	up
HCN2	1.23	up
MAPK7	1.23	up
TNFAIP2	1.23	up
AC099850	1.23	up
SERPINB5	1.23	up
ANXA1	1.23	up
FAM114A1	1.23	up
EPB41L1	1.22	up
DUS4L-BCAP29	1.22	up
AL390719	1.22	up
ARL8A	1.22	up
IGF2	1.22	up
TMEM159	1.21	up
EFNA2	1.21	up
FBLIM1	1.21	up
PDP1	1.21	up

B3GALT4	1.20	up
AL512274	1.20	up
AC025048	1.20	up
ADIRF	1.20	up
SPATA2L	1.20	up
JAG2	1.20	up
STARD9	1.20	up
LINC00514	1.20	up
CDR2L	1.20	up
RNF19B	1.19	up
C11orf80	1.19	up
DISP2	1.19	up
PRRG4	1.19	up
PWWP2B	1.19	up
ALDH1L2	1.19	up
SLC2A6	1.19	up
FAM89B	1.19	up
SLC9A3R2	1.18	up
PLXNA2	1.18	up
PMM1	1.18	up
UNC13D	1.18	up
DDX60L	1.18	up
KATNAL1	1.18	up
PEAR1	1.18	up
ZNF296	1.18	up
EHBP1L1	1.17	up
PRR34-AS1	1.16	up
CRYBG1	1.16	up
CELSR1	1.16	up
MEI1	1.15	up
DNAJB9	1.15	up
APAF1	1.14	up
MT1F	1.14	up
PTK6	1.14	up
TWIST2	1.14	up
DKK1	1.14	up
GALNT3	1.14	up
MYORG	1.13	up
ARHGAP27	1.13	up
APOL6	1.13	up
TMEM255B	1.13	up
CDS1	1.13	up
CYB561	1.13	up
CDA	1.13	up

CTSS	1.12	up
CACNA1C	1.12	up
ENTPD1	1.12	up
ZNF846	1.12	up
VSIR	1.12	up
PLAUR	1.12	up
HELZ2	1.12	up
AL928654	1.12	up
UPP1	1.11	up
GPC1	1.11	up
USP35	1.11	up
LGALS1	1.11	up
VILL	1.11	up
C19orf33	1.11	up
WDR47	1.10	up
PIM1	1.10	up
MYO1C	1.10	up
PLLP	1.10	up
NBPF25P	1.10	up
RPS26P6	1.10	up
S100P	1.10	up
TCF19	1.09	up
HRAS	1.09	up
RAB6C	1.09	up
PNPLA7	1.09	up
COL6A2	1.09	up
ARHGAP23	1.09	up
MPP3	1.08	up
EFNA4	1.08	up
CAV2	1.08	up
IRF5	1.08	up
ATXN7	1.08	up
DNAJB1	1.08	up
TRPV2	1.08	up
CENPO	1.08	up
JDP2	1.08	up
UNC13A	1.08	up
CCNYL1	1.08	up
KCTD10	1.08	up
CC2D1B	1.07	up
PRSS22	1.07	up
RIMS3	1.07	up
KLF11	1.06	up
SMAD7	1.06	up

CCDC9B	1.06	up
TMEM92	1.06	up
MICAL1	1.05	up
RBM24	1.05	up
AGAP2-AS1	1.05	up
CCDC24	1.05	up
PLEKHN1	1.05	up
BLVRA	1.05	up
CYP2S1	1.04	up
TRIM47	1.04	up
ZDHHC2	1.04	up
HYAL3	1.04	up
KLHL35	1.04	up
CSF2	1.04	up
CYTH3	1.04	up
S100A14	1.03	up
PER3	1.03	up
TNFAIP1	1.03	up
TRNAU1AP	1.03	up
UBE2K	1.03	up
KLF13	1.03	up
CALHM2	1.03	up
FUT2	1.03	up
MSLN	1.03	up
LAMB3	1.03	up
S100A11	1.03	up
ZC2HC1A	1.03	up
C15orf65	1.02	up
KIAA1841	1.02	up
MELTF	1.02	up
SH2D3A	1.02	up
CDKN1A	1.02	up
MMP14	1.02	up
IQGAP1	1.01	up
HMOX1	1.01	up
AC009065	1.01	up
CLDN7	1.01	up
CRMP1	1.01	up
SLC30A4	1.01	up
SNCG	1.01	up
CLCN6	1.00	up
DOP1B	1.00	up
CACNB3	1.00	up
IL17RD	1.00	up

RAB11FIP5	1.00	up
ANXA2	1.00	up
EXTL2	-1.00	down
AL137003	-1.00	down
CYFIP2	-1.00	down
MINCR	-1.00	down
C2orf15	-1.00	down
MEIS2	-1.00	down
KHDC4	-1.01	down
KCNMB4	-1.01	down
CILK1	-1.01	down
AP002360	-1.01	down
RALGAPA2	-1.01	down
ZNF211	-1.01	down
TCTE3	-1.01	down
AC129492	-1.01	down
KANTR	-1.01	down
DARS-AS1	-1.01	down
COQ8A	-1.01	down
MYT1	-1.02	down
CPLX2	-1.02	down
SNHG4	-1.02	down
HIPK2	-1.02	down
AC016876	-1.02	down
SLC5A3	-1.02	down
AL353763	-1.02	down
RBM26-AS1	-1.03	down
NR4A2	-1.03	down
B4GAT1	-1.03	down
MKI67	-1.03	down
PGAP1	-1.03	down
ASGR1	-1.03	down
ADGRL1	-1.03	down
FASLG	-1.03	down
ARID5B	-1.04	down
SCD5	-1.04	down
PTBP2	-1.04	down
ARL17B	-1.04	down
ZNF214	-1.04	down
ANKRD10	-1.04	down
PDGFRB	-1.04	down
TMEM161B-AS1	-1.04	down
RNF157	-1.04	down
TCAF2	-1.04	down

SLC9A3-AS1	-1.04	down
TMEM99	-1.04	down
ZNF213-AS1	-1.05	down
LINC02404	-1.05	down
AC004540	-1.05	down
FHOD1	-1.05	down
AL118516	-1.05	down
IFT27	-1.05	down
IGFL4	-1.05	down
SHC3	-1.06	down
PRCP	-1.06	down
AC104958	-1.06	down
RAD51AP1	-1.06	down
FZD3	-1.06	down
PLPP3	-1.06	down
PEX11A	-1.06	down
SUZ12	-1.06	down
OSR2	-1.06	down
TYSND1	-1.06	down
NDRG4	-1.06	down
SPATA12	-1.07	down
CDKN2C	-1.07	down
ZNF248	-1.07	down
SLCO5A1	-1.07	down
AL121759	-1.08	down
LINC01560	-1.08	down
ZNF789	-1.08	down
OSGEPL1	-1.08	down
ZNF704	-1.08	down
PPM1H	-1.08	down
BAIAP3	-1.08	down
LGR5	-1.08	down
MTERF2	-1.08	down
TMEM145	-1.09	down
ASB2	-1.09	down
KLLN	-1.09	down
INTS6-AS1	-1.09	down
VWA2	-1.09	down
PHF8	-1.09	down
LINC02418	-1.09	down
HAS2	-1.09	down
AC067750	-1.09	down
AC138409	-1.10	down
RASSF4	-1.10	down

KRT40	-1.10	down
CDCA7L	-1.10	down
SATB1	-1.10	down
TNC	-1.10	down
ZNF853	-1.10	down
C11orf71	-1.10	down
SLC7A8	-1.10	down
RPL32P3	-1.11	down
AC078846	-1.11	down
HOXA11-AS	-1.11	down
ST3GAL3	-1.11	down
DFFB	-1.11	down
AL359258	-1.11	down
EGR2	-1.11	down
AP005329	-1.11	down
MHENCR	-1.11	down
SLC27A2	-1.11	down
FAM214A	-1.12	down
PLOD2	-1.12	down
GABPB1-IT1	-1.12	down
AC007277	-1.12	down
PRPS2	-1.12	down
AL138479	-1.12	down
MRM1	-1.12	down
SP9	-1.12	down
PIF1	-1.12	down
CHST11	-1.12	down
STX18-AS1	-1.12	down
ZFAND4	-1.13	down
GSDMB	-1.13	down
TMPO-AS1	-1.13	down
GAS6-AS1	-1.13	down
RIBC2	-1.13	down
C1GALT1C1L	-1.14	down
TRIB2	-1.14	down
GABRA2	-1.14	down
PAXIP1-AS1	-1.14	down
AC069120	-1.14	down
EGLN3	-1.14	down
NPTXR	-1.14	down
AC097448	-1.14	down
BMP4	-1.15	down
ZNF436-AS1	-1.15	down
TMEM8B	-1.15	down

NBR2	-1.15	down
KLK3	-1.15	down
TFPI	-1.16	down
TMEM158	-1.16	down
NUPR1	-1.16	down
MYH10	-1.16	down
ZNF771	-1.16	down
BTD	-1.16	down
AC022211	-1.16	down
SBNO1-AS1	-1.17	down
L3HYPDH	-1.17	down
WNK2	-1.17	down
CDK18	-1.17	down
EPHA3	-1.17	down
LRP1	-1.17	down
CASTOR3	-1.17	down
AL109615	-1.17	down
LTB4R	-1.17	down
FAM117B	-1.18	down
PMS2P4	-1.18	down
TPPP3	-1.18	down
PCMTD1	-1.18	down
ARHGEF25	-1.18	down
AC012615	-1.18	down
AC079414	-1.18	down
AC009061	-1.19	down
GALNT12	-1.19	down
RALY-AS1	-1.19	down
PCYOX1L	-1.19	down
ILF3-DT	-1.19	down
QKI	-1.19	down
PROM1	-1.20	down
AC016394	-1.20	down
CX3CL1	-1.20	down
LINC01089	-1.20	down
TMEM198B	-1.20	down
AC010168	-1.20	down
LNP1	-1.20	down
CHRNA1	-1.20	down
MKRN3	-1.21	down
AP000866	-1.21	down
TMEM144	-1.21	down
PPFIA3	-1.21	down
SBK1	-1.21	down

MEIS1	-1.21	down
TTC25	-1.21	down
GPR3	-1.22	down
LINC00659	-1.22	down
AC026402	-1.22	down
RORA	-1.22	down
EID2B	-1.22	down
NTRK2	-1.23	down
GOLGA8A	-1.23	down
CD72	-1.23	down
PVRIG2P	-1.23	down
RRAGD	-1.23	down
STRA6	-1.24	down
H3C15	-1.24	down
EMID1	-1.24	down
AL117382	-1.24	down
H2BC18	-1.24	down
ZNF493	-1.24	down
DNHD1	-1.24	down
AC007405	-1.24	down
VSNL1	-1.24	down
LINC01106	-1.24	down
MYH3	-1.24	down
UBR5-AS1	-1.25	down
CNNM2	-1.25	down
SYNGR1	-1.25	down
TMPRSS5	-1.25	down
LRP2BP	-1.25	down
METTL7A	-1.25	down
RCN3	-1.25	down
AC015813	-1.25	down
PRORSD1P	-1.25	down
PELI2	-1.25	down
H3C14	-1.26	down
ZSWIM4	-1.26	down
H2BU1	-1.26	down
UCN2	-1.26	down
AC083843	-1.26	down
SMOC2	-1.26	down
DNAJC3-DT	-1.27	down
INPP5J	-1.27	down
DNAJB4	-1.27	down
GBGT1	-1.27	down
SESN3	-1.27	down

TRIM45	-1.28	down
AC018865	-1.28	down
AC006449	-1.28	down
AP002478	-1.28	down
HNF4G	-1.28	down
SLAMF9	-1.29	down
VASN	-1.29	down
TMEM63C	-1.29	down
AC241377	-1.29	down
AL365181	-1.30	down
BCL11B	-1.31	down
KDM7A-DT	-1.31	down
TTC30B	-1.31	down
AL355488	-1.31	down
SLC9A5	-1.31	down
AL132712	-1.31	down
WIPF2	-1.31	down
ANKRD10-IT1	-1.32	down
THAP6	-1.32	down
AC009113	-1.32	down
TRIM71	-1.32	down
AL133367	-1.32	down
AC068205	-1.32	down
URB1-AS1	-1.32	down
ADAMTSL2	-1.33	down
TET1	-1.33	down
LRG1	-1.33	down
DNM3	-1.33	down
TMEM97	-1.33	down
RGS2	-1.34	down
AL021707	-1.34	down
SOX2	-1.34	down
AC008966	-1.34	down
RASL10B	-1.34	down
LASTR	-1.34	down
PAXBP1-AS1	-1.34	down
H2BC19P	-1.34	down
AC132872	-1.35	down
NLGN2	-1.35	down
SLC25A27	-1.35	down
SLC46A3	-1.35	down
AC241585	-1.36	down
ADAM22	-1.36	down
P3H3	-1.36	down

KIF7	-1.36	down
ARIH2OS	-1.36	down
C8orf44	-1.36	down
LINC02261	-1.37	down
MCTP1	-1.37	down
HSD52	-1.37	down
FAM234B	-1.37	down
PRSS2	-1.37	down
AC097639	-1.38	down
SPATA6	-1.39	down
CCL24	-1.39	down
AC025774	-1.39	down
CTSH	-1.39	down
FAM222A	-1.39	down
ZNF383	-1.40	down
ZNRD1ASP	-1.40	down
SLC45A1	-1.40	down
COL1A1	-1.40	down
NGFR	-1.40	down
BHLHE41	-1.40	down
AC104596	-1.40	down
VWCE	-1.40	down
PLA2G4D	-1.40	down
MIR34AHG	-1.41	down
RASSF5	-1.41	down
CCDC80	-1.41	down
ZNF814	-1.41	down
AC016168	-1.41	down
TNS1	-1.42	down
MILR1	-1.42	down
LMO2	-1.42	down
ASB4	-1.42	down
RIMKLA	-1.42	down
AL133215	-1.43	down
NIPBL-DT	-1.43	down
VWA5A	-1.43	down
DNAJB5	-1.43	down
PLEKHH2	-1.43	down
GABRE	-1.43	down
DIPK1B	-1.44	down
GOLGA2P5	-1.44	down
WNT6	-1.44	down
ZNF215	-1.44	down
UQCC1	-1.45	down

SCG2	-1.45	down
ATXN1-AS1	-1.45	down
TRPV4	-1.46	down
NRGN	-1.46	down
TAGAP	-1.47	down
BMF	-1.47	down
ALDH1A2	-1.47	down
ARRDC3-AS1	-1.47	down
CBX7	-1.48	down
CLGN	-1.48	down
MEF2B	-1.48	down
AC104024	-1.49	down
TMEM45A	-1.49	down
SGSM1	-1.49	down
GVQW3	-1.49	down
NKX2-1	-1.49	down
AC068870	-1.49	down
TBX6	-1.50	down
FAM13C	-1.50	down
ZNF345	-1.50	down
NREP	-1.50	down
FXYD2	-1.50	down
TTC30A	-1.51	down
CCDC74A	-1.51	down
AC005224	-1.51	down
EIF2AK3-DT	-1.51	down
ITPR2	-1.51	down
PTGES2-AS1	-1.52	down
BCAR3-AS1	-1.52	down
LEKR1	-1.52	down
AL513534	-1.52	down
AL353150	-1.52	down
SSTR1	-1.53	down
MFSD4B	-1.53	down
FER1L4	-1.53	down
ENTPD1-AS1	-1.53	down
AC092338	-1.54	down
SLC16A10	-1.54	down
B3GAT1	-1.55	down
LRP4	-1.55	down
MXI1	-1.55	down
AOAH	-1.55	down
FSD1	-1.55	down
GPR155	-1.56	down

AL009179	-1.56	down
DPY19L2P2	-1.56	down
AC092164	-1.56	down
FAM13A	-1.56	down
Z95115	-1.57	down
AC125616	-1.57	down
AC121761	-1.57	down
TTYH2	-1.58	down
CD8B	-1.59	down
AL359915	-1.59	down
CRABP2	-1.60	down
SARDH	-1.60	down
SLC38A3	-1.60	down
CREB5	-1.62	down
AC004585	-1.62	down
SFRP5	-1.62	down
ZNF610	-1.63	down
ATP6V0A4	-1.63	down
JAKMIP2-AS1	-1.63	down
CASC15	-1.63	down
AC245060	-1.63	down
PLB1	-1.64	down
AC119403	-1.64	down
ZNF608	-1.64	down
AK9	-1.65	down
AC104389	-1.65	down
AL133410	-1.65	down
BDNF-AS	-1.65	down
DLX6	-1.67	down
ZNF710-AS1	-1.67	down
TBC1D10C	-1.67	down
TNFRSF13C	-1.67	down
ANGPT2	-1.67	down
CD6	-1.67	down
FILIP1L	-1.68	down
AC013565	-1.69	down
ATP2A1	-1.69	down
AC007191	-1.69	down
ZNF587B	-1.69	down
AC245100	-1.69	down
PRUNE2	-1.70	down
MYH7B	-1.70	down
AC099522	-1.70	down
AC011700	-1.71	down

SDAD1P1	-1.71	down
SLCO4A1-AS1	-1.71	down
ZNF33B	-1.71	down
PRAC2	-1.72	down
COL8A1	-1.72	down
MME	-1.72	down
UMAD1	-1.73	down
AC239868	-1.73	down
PDK4	-1.73	down
AL109615	-1.73	down
KCTD21-AS1	-1.73	down
AC021683	-1.73	down
AL162458	-1.74	down
ANKRD34A	-1.74	down
AL035587	-1.74	down
AP002852	-1.74	down
NEDD9	-1.75	down
KRT5	-1.75	down
AC188617	-1.75	down
AL133325	-1.76	down
TRBJ2-5	-1.76	down
ALDH5A1	-1.78	down
SLC30A3	-1.78	down
SPTY2D1OS	-1.78	down
DISC1	-1.78	down
SIT1	-1.79	down
ASH1L-AS1	-1.80	down
RASL11B	-1.80	down
ANKRD22	-1.80	down
RNF183	-1.80	down
ABHD1	-1.80	down
LINC01622	-1.81	down
AC004890	-1.81	down
AC073896	-1.82	down
LINC01226	-1.82	down
RTCA-AS1	-1.83	down
KCNJ2	-1.83	down
PSD2	-1.84	down
AC016705	-1.84	down
AL138960	-1.84	down
ACTA2	-1.84	down
LINC01534	-1.85	down
TNFSF10	-1.85	down
MIR181A2HG	-1.86	down

EFCAB6	-1.86	down
ISX	-1.86	down
SLC2A4	-1.87	down
TSPOAP1	-1.87	down
SSTR2	-1.88	down
HOTAIR	-1.88	down
LINC01424	-1.89	down
APLN	-1.89	down
LOX	-1.89	down
AC130650	-1.90	down
PCAT6	-1.90	down
AC017083	-1.90	down
TSPAN10	-1.91	down
VWA5B2	-1.91	down
AC006511	-1.92	down
CCR7	-1.92	down
LINC01238	-1.93	down
LINC01346	-1.93	down
ANPEP	-1.94	down
FZD2	-1.94	down
THBS1	-1.94	down
FAM3B	-1.94	down
LRFN1	-1.95	down
AC026336	-1.96	down
TUBBP6	-1.96	down
AP000311	-1.97	down
MROCKI	-1.97	down
PLA1A	-1.97	down
ANTXR1	-1.97	down
SHISA9	-1.97	down
AL353622	-1.98	down
AC124016	-1.98	down
TRBJ2-7	-1.98	down
NBEA	-1.99	down
PMF1-BGLAP	-2.00	down
STX17	-2.01	down
BCL11A	-2.01	down
AC241585	-2.01	down
HIF1A-AS3	-2.02	down
SPARC	-2.02	down
UCN	-2.03	down
RAB26	-2.03	down
AL390067	-2.04	down
IGSF9	-2.04	down

LIPC	-2.05	down
AL391684	-2.05	down
SEMA3G	-2.05	down
ZNF570	-2.05	down
BNIP3	-2.06	down
AL163953	-2.06	down
BCDIN3D-AS1	-2.06	down
MPP2	-2.07	down
GALNT16	-2.07	down
H4-16	-2.10	down
AC068580	-2.10	down
AC015813	-2.11	down
KCNH6	-2.12	down
LINC01348	-2.13	down
LINC00634	-2.14	down
AL590652	-2.15	down
AP004608	-2.16	down
SRGAP3	-2.17	down
AF064858	-2.19	down
MYLK	-2.21	down
DACH1	-2.21	down
AC016355	-2.22	down
RASGRF1	-2.23	down
COL3A1	-2.23	down
ADGRL2	-2.23	down
NRN1	-2.24	down
AC241952	-2.24	down
AC138894	-2.25	down
CCT7P2	-2.25	down
ATAD3C	-2.26	down
PRICKLE2	-2.28	down
AC138904	-2.28	down
KRT39	-2.28	down
ERV3-1	-2.29	down
DIXDC1	-2.32	down
AC087762	-2.34	down
NRROS	-2.35	down
SYT12	-2.35	down
FLG-AS1	-2.35	down
PURPL	-2.36	down
AL355075	-2.36	down
COL1A2	-2.36	down
BCHE	-2.38	down
INHA	-2.40	down

AC007541	-2.41	down
AC114811	-2.42	down
PPFIA4	-2.43	down
MBL1P	-2.43	down
ZNF8-ERVK3-1	-2.43	down
GPR162	-2.44	down
LINC01132	-2.44	down
VANGL2	-2.45	down
TENT5C	-2.46	down
TTC41P	-2.46	down
MRPS30-DT	-2.47	down
AL160269	-2.47	down
CXCR4	-2.48	down
SLC2A13	-2.51	down
TMEM121	-2.58	down
TRIM73	-2.59	down
TRIM9	-2.60	down
ZNF419	-2.60	down
SEC31B	-2.61	down
AP000553	-2.62	down
PCDH19	-2.63	down
SMO	-2.64	down
CAMKV	-2.65	down
SOX5	-2.65	down
LIMD1-AS1	-2.66	down
PPM1L	-2.67	down
AL137802	-2.67	down
SEC14L5	-2.70	down
AC116535	-2.71	down
SCN3A	-2.74	down
CRYGN	-2.74	down
DBH-AS1	-2.74	down
CD37	-2.76	down
AC091132	-2.79	down
PTPRS	-2.81	down
CD5	-2.84	down
AC026523	-2.85	down
HCG25	-2.90	down
SYNPO2	-2.91	down
TRBV30	-2.94	down
KCNIP2-AS1	-2.95	down
TF	-2.96	down
ADAM11	-2.96	down
VAC14-AS1	-3.00	down

GOLGA6L7	-3.07	down
TPSAB1	-3.08	down
ST18	-3.09	down
AC116366	-3.12	down
PCDHGA10	-3.15	down
LINC01237	-3.19	down
TGFB2	-3.22	down
CCND2	-3.22	down
ERLEC1P1	-3.23	down
AP006748	-3.26	down
AC091812	-3.27	down
LINC01134	-3.29	down
AC104823	-3.33	down
BCAT1	-3.58	down
AP000526	-3.59	down
OGDHL	-3.67	down
AL391825	-3.68	down
KRTAP3-2	-3.79	down
AC005041	-3.80	down
UCHL1	-3.95	down
AL138789	-4.03	down
AL513190	-4.08	down
DBH	-4.08	down
POU3F2	-4.09	down
MT3	-4.12	down
ADAMTS18	-4.19	down
AC015909	-4.20	down
SPDYE2B	-4.23	down
ZP1	-4.23	down
AP001486	-4.25	down
THY1	-4.26	down
H1-9P	-4.28	down
LINC00960	-4.32	down
CA12	-4.47	down
ABCA10	-4.51	down
AC084262	-4.54	down
SLC19A3	-4.66	down
AC114490	-4.72	down
PPP4R4	-4.73	down
Z69890	-4.80	down
KIF19	-4.94	down
AC010654	-4.98	down
AC004080	-5.13	down
HTRA3	-5.45	down

SPATA48	-5.49	down
ADORA2A	-5.51	down
AC008946	-5.74	down
LY6G6F	-6.00	down
SPP1	-6.29	down
AC007663	-6.43	down
ARHGAP28	-7.01	down
AL160272	-7.11	down
LAMA1	-7.29	down
KCNK12	-7.31	down
AP000356	-7.61	down
GAPDHP22	-7.64	down
POU2AF1	-7.81	down
AC011462	-7.97	down
ITGBL1	-8.05	down
GLP2R	-8.07	down
GPR137C	-8.27	down
CERKL	-8.40	down
FBLN7	-8.41	down
PCDHGA12	-8.48	down
MATN2	-8.51	down
SSC5D	-8.52	down
NCMAP	-8.53	down
ZIK1	-8.56	down
AC119673	-8.61	down
SIAH2-AS1	-8.69	down
DSCAML1	-8.72	down
CDH2	-8.79	down
DEGS2	-8.85	down
FREM1	-8.98	down
CXXC4	-9.04	down
DRC7	-9.05	down
NPHP3-ACAD11	-9.11	down
EFEMP1	-9.39	down
SPOCK1	-9.39	down
AC063919	-9.49	down
AC012508	-9.52	down
FAM163B	-9.56	down
CYP4F62P	-9.61	down
HS3ST4	-9.65	down
NODAL	-9.70	down
AC005229	-9.88	down
CSPG5	-9.91	down
YPEL4	-9.95	down

CD14	-10.01	down
AC097532	-10.15	down
AC104809	-10.32	down
AC104088	-10.34	down
EDN2	-10.40	down
AC006030	-10.52	down
AC112128	-10.53	down
AC020915	-10.67	down
AL021308	-10.70	down
AL353807	-10.85	down
LRRC26	-10.90	down
OGFOD1P1	-10.94	down
AC091178	-10.97	down
FAM138C	-11.02	down
LINC02158	-11.06	down
AL392089	-11.13	down
AC026471	-11.17	down
AC068413	-11.29	down
TPSB2	-11.45	down
AL021920	-11.50	down
AC015819	-11.52	down
BX649632	-11.56	down
AL445222	-11.59	down
AC002310	-11.62	down
AC055717	-12.31	down
AC051619	-12.44	down
CERS1	-12.52	down
AL137779	-12.64	down
AC104076	-12.74	down
AC010422	-12.84	down
AC024560	-12.85	down
AL133380	-12.90	down
PCDHGB5	-13.01	down
AL121900	-13.71	down
PDCD6-AHRR	-15.05	down
ISY1-RAB43	-15.28	down
FMC1-LUC7L2	-16.58	down

Table S4. Kyoto Encyclopedia of Genes and Genomes (KEGG) analysis

Pathway name	S gene number	TS gene number	B gene number	TB gene number	*P* value
Proteoglycans in cancer	22	324	187	6424	0.000163
Protein digestion and absorption	12	324	77	6424	0.000420
Hematopoietic cell lineage	10	324	64	6424	0.001233
Transcriptional misregulation in cancer	18	324	168	6424	0.001900
Basal cell carcinoma	9	324	60	6424	0.002864
Focal adhesion	18	324	181	6424	0.004293
Gap junction	10	324	76	6424	0.004550
Jak-STAT signaling pathway	13	324	121	6424	0.007656
Axon guidance	16	324	166	6424	0.009262
ECM-receptor interaction	9	324	72	6424	0.009721
PI3K-Akt signaling pathway	24	324	296	6424	0.013861
Human papillomavirus infection	24	324	301	6424	0.016726
Cytokine-cytokine receptor interaction	17	324	195	6424	0.018937
MicroRNAs in cancer	17	324	197	6424	0.020722
Phospholipase D signaling pathway	12	324	125	6424	0.023266
Ras signaling pathway	17	324	204	6424	0.027997
Glycosphingolipid biosynthesis - globo and isoglobo series	3	324	14	6424	0.030570
MAPK signaling pathway	21	324	270	6424	0.030912
Malaria	5	324	36	6424	0.032993
Prolactin signaling pathway	7	324	62	6424	0.035393
